# *Prochlorococcus marinus* responses to light and oxygen

**DOI:** 10.1371/journal.pone.0307549

**Published:** 2024-07-22

**Authors:** Mireille Savoie, Aurora Mattison, Laurel Genge, Julie Nadeau, Sylwia Śliwińska-Wilczewska, Maximilian Berthold, Naaman M. Omar, Ondřej Prášil, Amanda M. Cockshutt, Douglas A. Campbell

**Affiliations:** 1 Department of Biology, Mount Allison University, Sackville, New Brunswick, Canada; 2 Department of Chemistry and Biochemistry, Mount Allison University, Sackville, New Brunswick, Canada; 3 Department of Biology, University of British Columbia, Vancouver, British Columbia, Canada; 4 Fisheries and Oceans Canada, Ecosystems Management Branch, Dartmouth, Nova Scotia, Canada; 5 Institute of Oceanography, University of Gdansk, Gdynia, Poland; 6 Laboratory of Photosynthesis, Institute of Microbiology, Center Algatech, Trebon, Czech Republic; 7 Department of Chemistry, St. Frances Xavier University, Antigonish, Nova Scotia, Canada; Bigelow Laboratory for Ocean Sciences, UNITED STATES OF AMERICA

## Abstract

*Prochlorococcus marinus*, the smallest picocyanobacterium, comprises multiple clades occupying distinct niches, currently across tropical and sub-tropical oligotrophic ocean regions, including Oxygen Minimum Zones. Ocean warming may open growth-permissive temperatures in new, poleward photic regimes, along with expanded Oxygen Minimum Zones. We used ocean metaproteomic data on current *Prochlorococcus marinus* niches, to guide testing of *Prochlorococcus marinus* growth across a matrix of peak irradiances, photoperiods, spectral bands and dissolved oxygen. MED4 from Clade HLI requires greater than 4 h photoperiod, grows at 25 μmol O_2_ L^-1^ and above, and exploits high cumulative diel photon doses. MED4, however, relies upon an alternative oxidase to balance electron transport, which may exclude it from growth under our lowest, 2.5 μmol O_2_ L^-1^, condition. SS120 from clade LLII/III is restricted to low light under full 250 μmol O_2_ L^-1^, shows expanded light exploitation under 25 μmol O_2_ L^-1^, but is excluded from growth under 2.5 μmol O_2_ L^-1^. Intermediate oxygen suppresses the cost of PSII photoinactivation, and possibly the enzymatic production of H_2_O_2_ in SS120, which has limitations on genomic capacity for PSII and DNA repair. MIT9313 from Clade LLIV is restricted to low blue irradiance under 250 μmol O_2_ L^-1^, but exploits much higher irradiance under red light, or under lower O_2_ concentrations, conditions which slow photoinactivation of PSII and production of reactive oxygen species. In warming oceans, range expansions and competition among clades will be governed not only by light levels. Short photoperiods governed by latitude, temperate winters, and depth attenuation of light, will exclude clade HLI (including MED4) from some habitats. In contrast, clade LLII/III (including SS120), and particularly clade LLIV (including MIT9313), may exploit higher light niches nearer the surface, under expanding OMZ conditions, where low O_2_ relieves the stresses of oxidation stress and PSII photoinhibition.

## Introduction

### *Prochlorococcus marinus* diversity

*Prochlorococcus marinus*, a genus of Cyanobacteria, is the smallest known photosynthetic prokaryote, with cell diameters ranging from 0.5 to 0.7 μm [1]. Despite its small cell size, *P*. *marinus* contribute 13 to 48% of net primary production in oligotrophic oceans, corresponding to about 30% of global oxygen production [2]. *Prochlorococcus marinus* growth is currently limited to between latitudes of 40°N to 40°S in open ocean waters, from surface to 300 m depth, thus spanning 3 orders of magnitudes of light irradiance [1,2].

*Prochlorococcus marinus* comprises many strains, organized into clades, defined by 16S-23S intergenic transcribed ribosomal sequence signatures [3]. The clades inhabit distinct ecological niches [4], originally defined as High-Light (HL) or Low-Light (LL). Only 5 out of 12 known *P*. *marinus* genetic clades have cultured representatives to date; HLI, HLII, LLI, LLII/III and LIV [5–7]. Current niches of *P*.*marinus* strains span ocean water columns [2,8,9] and extend into regions with low dissolved oxygen concentrations [10–14].

Low-Light clades thrive in deeper ocean waters, extending beyond 200 m in depth [2], where only ~1% of the surface irradiance penetrates, primarily in the blue (450 nm) to green (520 nm) spectral range [15]. Clade LLI includes cultured strain NATL2A, which prefers moderate irradiances typical of between 30 and 100 m depth. Clades LLII and LLIII, including cultured strain SS120, are grouped together as early branching phylogenetic lineage in the *P*. *marinus* radiation, with a preference for low light. Clade LLIV, including cultured strain MIT9313, falls near the base of the *P*. *marinus* radiation, and has been characterized by preference for low light, typical of depths from 120 m to 200 m [2]. Clade LLIV members are, as yet, the only cultured *P*. *marinus* strains to have been found in Oxygen Minimum Zones (OMZ). Some, as yet, uncultured *P*. *marinus* strains in clades LLV and LLVI also thrive in OMZ of the subtropical Atlantic and Pacific Oceans, where dissolved oxygen concentrations [O_2_] can be less than 20 μM [11–14,16]. *Prochlorococcus marinus* LL ecotypes may indeed dominate the phytoplankton within OMZ [10,12,13], where they may be net O_2_ consumers [17].

High-Light clades are more recently branching lineages, with reduced genome sizes in comparison to LL clades. High-Light clades are typically dominant picophytoplankters in near-surface, oligotrophic waters, characterized by high light levels and subdivided into clades based on iron adaptation [18–20]. Clade HLI, represented by cultured strain MED4, is adapted to high iron, and low temperatures, and originated from 5 m depth in the Mediterranean Sea [2]. Clade HLII, adapted to high iron, and high temperatures, is the most abundant *P*. *marinus* clade in the North Atlantic and North Pacific Oceans, often constituting over 90% of the total population [2], and are most numerous around 50 m depth [2]. Clade HLIII/IV is adapted to low iron [18–20].

*Prochlorococcus marinus* clades are nonetheless found in environments beyond their optimal habitats. HL clades inhabit depths overlapping with LL ecotypes [19,21,22], while LL clades can occupy regions in OMZ at depths shallower than 40 m [12], exploiting ambient light levels above what LL clades were thought to tolerate.

### *Prochlorococcus marinus* and changing niches

Our changing climate is rapidly altering conditions for these specialized clades of marine picophytoplankton. Predictions indicate a net global increase of *P*. *marinus* cell abundances of 29% [23], along with poleward latitudinal shifts of at least 10° in marine phytoplankton niches by the end of this century [24] in response to warming waters, with increases in *P*. *marinus* of approximately 50% in the more poleward regions of their distributions. Follett *et al*. [25], however, model interactions of heterotrophic bacteria that may influence latitudinal expansions of *P*. *marinus*.

Near the equator, photoperiod remains nearly constant at the ocean surface, approximately 12 hours (h) of daylight and 12 h of darkness throughout the year. The effective length of the photoperiod does, however, attenuate with depth as dawn and dusk light at depth drops below levels needed for biological processes. As *P*. *marinus* potentially expands its temperature-permissive niches poleward into temperate regions [23,24], it will encounter more pronounced seasonal variations in photoperiod regimes both at surface and at depth, with potentially complex effects upon viable growth niches [26,27]. For example, Vaulot *et al*. [28] showed that *P*. *marinus* replication of DNA occurs in the afternoon, while cell division occurs at night. To our knowledge, no study has as yet addressed *P*. *marinus* growth rate responses in relation to a range of photoperiods.

Climate change is also rapidly changing ocean chemistry. By the end of this century, surface ocean pH is projected to decline by 0.1 to 0.4 due to projected increases in carbon dioxide concentrations [29]. Moreover, substantial changes in the global water cycle, leading to extensive changes in worldwide precipitation patterns, are affecting ocean salinity levels on a global scale, and ice melts due to rising temperatures are impacting salinity levels in the Arctic and Northwest Atlantic oceans [30]. Increasing sea temperatures are also causing decreases in [O_2_] across global oceans [31], particularly toward the poles [32]. Warmer ocean waters decrease oxygen solubility at the surface, and increase stratification, which in turn decreases oxygen mixing downwards by ocean currents [29]. Models predict that OMZ in the Pacific and Indian Oceans are expanding [29,33], although the cores of the OMZ, where the oxygen levels are lowest, may actually contract [33].

We used the Ocean Protein Portal (OPP; https://www.oceanproteinportal.org/) [34] to analyze the distribution of proteins from clades of *P*. *marinus* in samples taken across a range of [O_2_] and depth, which in turn correlates to depth attenuated peak light at the site of sampling. In parallel we analyzed the growth rate and physiological responses of representative strains from three clades of *P*. *marinus* under a matrix of [O_2_], light levels, photoperiods and spectral waveband ranges, to approximate eco-physiological conditions representative of current and hypothetical future ocean zones. *Prochlorococcus marinus* MED4, a clade HLI strain, was isolated near the ocean surface (5m depth) of the Mediterranean Sea where [O_2_] is near saturation, light levels are high and spectral bias from full solar irradiance is minimal. *Prochlorococcus marinus* SS120, a clade LLII/III strain, was isolated from the Sargasso Sea at a depth of 120 m, while *P*. *marinus* MIT9313, a clade LLIV strain, was isolated from the North Atlantic Gulf Stream at a depth of 135 m [35]. At these depths, light attenuation and spectral shifts occur, resulting in low blue light, while [O_2_] varies from near-surface saturation levels to decreased concentrations, but does not necessarily decrease systematically with depth [36].

Photosynthetic organisms absorb light energy within the Photosynthetically Active Radiation (PAR) range, 350 to 700 nm, for photosynthesis [37]. Photosynthetically Usable Radiation (PUR) represents the fraction of PAR that can be absorbed by the pigments of a given photosynthetic organism [37], taking into account the specific spectral wavebands these pigments absorb. *Prochlorococcus marinus* Pcb light-harvesting complexes show an absorption maxima of 442 nm for divinyl chlorophyll *a* and 478 nm for divinyl chlorophyll *b* [38] allowing *P*. *marinus* to efficiently harvest blue light in the 400 nm to 500 nm range [37] corresponding to blue spectral wavelengths prevailing in deep ocean habitats [15]. In *P*. *marinus* small cell diameters, from 0.5 to 0.7 μm [1], and simple cell structures, minimize the complication of pigment package effect or intracellular self-shading [39] contributing to efficient optical absorption, although photosynthetic efficiency may vary among clades [35,40].

Given the different spectral light regimes typical of the niches of different ecotypes, expressing growth rates in terms of cumulative diel PUR might simplify different photoperiods, PAR levels, and spectral bands into a common parameter, making growth rate comparisons across strains and different oxygen levels more accessible. We aimed to detect whether growth rates are driven simply by cumulative diel PUR, or whether specific photoperiods, PAR levels or spectral bands have independent, albeit interacting, effects on growth. We therefore analyzed growth rates in terms of cumulative diel PUR.

We discuss our findings in relation to analyses of genomic sequences [41] across clades of *P*. *marinus*, showing that differences in the expression and presence of genes encoding protein turnover, oxygen-dependent enzymes, and DNA repair enzymes, can explain the differential growth rate responses of strains under the matrix of light and [O_2_] conditions of this study.

## Materials and methods

### Metaproteomics

The Ocean Protein Portal is an open access online data repository (Woods Hole Oceanographic Institution, WHOI) of mass spectroscopy data on marine microbial proteins, sampled from various depths and locations worldwide [34]. We screened a subset of the OPP for proteins annotated as from *P*. *marinus* strains, to identify differential strategies employed by strains living at varying depths and oxygen levels within the marine water column. We focused on proteins mediating photosynthesis and protein metabolism from depths of 20 to 200 m below the ocean surface. The samples for metaproteomic analyses were collected from 7 locations in the tropical North Pacific Ocean along 150 W from 18 N of the equator between October 1, 2011 and October 25, 2011 during the voyage of the R/V Kilo Moana MetZyme expedition [42]; original datasets in the Biological and Chemical Oceanography Data Management Office (BCO-DMO) repository (https://www.bco-dmo.org/project/2236) [43]. Oxygen concentration levels at the location of sampling were recorded. The methodology for sample collection and peptide analysis are described by Saito *et al*. [44,45].

### Metaproteomics bioinformatic analyses

Metaproteomic datasets were obtained from the KM1128 entry in the BCO-DMO repository [43] accessed via the OPP in June 2019 at https://www.bco-dmo.org/dataset-deployment/730728. This dataset included biomass in the 0.2 to 3.0 micron size fractionated filter size as described in Saito *et al*. [44] where *P*. *marinus* is found. Datasets contained: i) Protein sequences and sample identification (ID) number; ii) Sample ID number, station, depth in meters below the surface the sample was collected at, best-hit BLASTP protein and species annotation and the corresponding Uniprot Entry number for the identified proteins; iii) Sample station depth and [O_2_]. The depth and [O_2_] (also from BCO-DMO at https://www.bco-dmo.org/dataset/646115/) were joined to protein sequence and BLASTP annotations by ID number, depth and station using *tidyverse* packages [46] running under R v4.1.3 and RStudio v2023.06.0 [47]. The resulting merged dataset was filtered for those *P*. *marinus* protein, detected from 0 to 300 m below the surface, annotated as a subunit of *P*. *marinus* chlorophyll binding proteins (Pcb); Photosystem II (PSII); Cytochrome b_6_f (Cytb_6_f); Photosystem I (PSI); NADPH Dehydrogenase (NDH); Plastoquinol Terminal Oxidase (PTOX); Plastocyanin (PC); Ferredoxin (Fd); Ribulose-1,5-bisphosphate oxygenase (RUBISCO); Adenosine triphosphate (ATP) Synthase; FtsH proteases (FtsH) or ribosomes. Detected peptides were re-annotated for consistency and labelled, where feasible, according to strain, clade, subunit and protein complex. Full protein sequences corresponding to detected proteins were obtained from UniProt (https://www.uniprot.org/) and analyzed in Molecular Evolution and Genetic Analyses X (MEGAX) software (https://www.megasoftware.net/). Sequences for proteins for each of the thirteen *P*. *marinus* strains identified in the dataset were aligned with MUSCLE using UPGMA cluster method and a lambda of 24 with a -2.9 gap open penalty and 1.20 hydrophobicity multiplier. Overall mean pairwise distance between protein sequences was determined using bootstrap variance estimation methods. Maximum likelihood phylogenetic trees were assembled using 1000 bootstrap replications with a 95% site coverage cut off. *Prochlorococcus marinus* FtsH isoform identities, and functions, were inferred by sequence comparisons to the characterized four isoforms of FtsH protease of *Synechocystis* sp. PCC6803 [48]. Data for each strain was plotted against depth and [O_2_] and sampling station.

When assessing the presence of a particular protein complex at a sampling location, the spectral counts were summed from all protein subunits from the protein complex, to increase probability of detection. As this data was acquired by survey proteomics (data dependent acquisition) rather than through targeted peptide approaches (e.g. parallel reaction monitoring), it is difficult to discern accuracies of strain assignment annotations, particularly as the proteins of interest in this study are highly conserved across strains [45]. The data is also limited because a peptide sequence was not determined unless there was already a known spectrum for that peptide in the SEQUEST database, hence some peptides of interest may not be identifiable. The MetZyme dataset used a deep paired metagenomic database (https://www.ebi.ac.uk/pride/archive/projects/PXD030684) to enable peptide-to-spectrum matching [44,49]. Furthermore, protein identifications were based on peptide-to-spectrum matching using SEQUESTHT within Proteome Discoverer software (Thermo) and spectral counts were enumerated using Scaffold software (Proteome Software) using a FDR of <0.1% on the peptide level as described in Saunders *et al*. [50]. While the accuracy of strain specific protein annotations are limited due to the high conservation of the target protein complexes, the two step approach of peptide-to-spectrum matching using deep paired metagenomics, does assign proteins at the level of clades.

### *Prochlorococcus marinus* culturing and experimental design

*Prochlorococcus marinus* remain challenging to culture at high densities or under fluctuating environments, partially due to their dependence upon mutualistic heterotrophic bacteria to detoxify reactive oxygen species [51,52]. MED4, SS120 and MIT9313 have been successfully cultured in laboratories [5,6], and used to show that ecotypic classifications correspond to biochemical differences among strains [48]. Three xenic cultures of *P*.*marinus* were obtained from Bigelow Labs, NCMA Maine, USA. MED4 (CCMP1986) is from High-Light adapted (HLI) clade; SS120 (CCMP1375) is from Low-Light adapted (LLII/III) clade; and MIT9313 (CCMP2773) is from Low-Light adapted (LLIV) clade. Cultures were maintained in incubators set to 22°C with an on/off light/dark cycle of 12 h. The PAR level for maintenance cultures reflected PAR in the source niche of the ecotype; MED4, of 160 μmol photons m^-2^ s^-1^ with illumination from STANDARD Products Inc. Cool White F24T5/41K/8/HO/PS/G5/STD, 24 watts, fluorescent bulbs; SS120 and MIT9313 at 30 μmol photons m^-2^ s^-1^ with illumination from Philips Cool White F14T5/841 Alto, 14 watts, fluorescent bulbs. To maintain active growth all strains were transferred weekly with 1 in 5 dilutions with Pro99 media [6] prepared with autoclaved artificial seawater [53].

Controlled growth rate experiments were then performed using MCMIX-OD or MC1000-OD PSI Multicultivators (S1 Fig; PSI, Drásov, Czech Republic). Each multicultivator individually controls 8 tubes at a common temperature of 22°C. Each tube containing 70 mL of Pro99 media was inoculated around mid day of the 12 h maintenance photoperiod with 10 mL of growing maintenance preculture, to reach a starting OD680 of approximately 0.020. The tubes containing the cultures were then placed in the Multicultivator water bath set at 22°C, sparged with the experimental [O_2_], and kept at low light until late afternoon. Cultures were then in the dark for 12 to 16 hr until the photoregime of a sinusoidal photoperiod commenced the following morning, reaching peak PAR at noon each day. Cultures thus took approximately 24 h to move gradually from maintenance photoregime to the peak PAR of the experimental photoregime. Cultures were grown for 7 to 14 days, until they reached stationary phase at OD680 of approximately 0.4 to 0.8 after approximately 5 generations of growth.

In a factorial matrix design, each tube was subject to an individual combination of sinusoidal photoperiod (4, 8, 12, 16 h); reaching a peak PAR (30, 90, 180 μmol photons m^-2^ s^-1^), with defined spectral bandwidth (White LED, 660 nm, 450 nm). [O_2_] levels (2.5 μM, 25 μM, 250 μM) were imposed by bubbling tubes with varying ratios of air and Nitrogen (N_2_), with consistent 0.05% of Carbon Dioxide (CO_2_) gas, delivered through a 0.2 μm sterile microfilter via a G400 gas mixing system (Qubit Systems Inc., Kingston, Ontario, Canada). [O_2_] *in situ* was verified using oxygen optodes (PyroScience, Germany) inserted into tubes for real-time measurements, with a temperature probe in the bath of the bioreactor to correct [O_2_] measures for temperature fluctuations. In addition, the Pyroscience software corrected [O_2_] based on the salinity of the media (32 ppt). The flow rate of the gas mixture was controlled, but variations in bubbling speed, PAR and culture density affected the [O_2_] achieved in each tube. A low [O_2_] of 0.5 μM—5 μM (reported as 2.5 μM hereafter), was achieved by sparging with a gas mixture containing 99.95% N_2_ and 0.05% CO_2_. An intermediate [O_2_] of 10–25 μM (reported hereafter as 25 μM) was achieved by sparging with a gas mixture containing 98.95% N_2_, 0.05% CO_2_ and 1% O_2_. A high O_2_ of 200 μM—280 μM (reported hereafter as 250 μM) was achieved by sparging with lab air (78% N_2_, 21% O_2_, 1% Ar and 0.05% CO_2_).

The full crossing of all factor levels would yield 4 x 3 x 3 x 3 = 108 treatments, x 3 strains for 324 possible combinations. Consistent absence of growth of some strains under some levels of photoperiod, PAR, or [O_2_] meant we completed 268 growth rate factor treatment combinations with 1 to 3 replicates.

*In situ* measurements of Optical Density (OD) 680 nm, a proxy for cell suspension density, cell size dependent scatter and cell chlorophyll content; and OD 720 nm, a proxy for cell suspension density and cell size dependent scatter, were recorded every 5 minutes over least 8 to 14 days, depending on the duration of the lag phase, if any.

Peak PAR of 180, 90 or 30 μmol photons m^-2^ s^-1^, and spectral wavebands (white LED full spectrum, 660 nm (red light), and 450 nm (blue light)) were chosen to approximate light levels and spectral colours spanning the vertical ocean water column, from near-surface to the lower euphotic zone depths. Photoperiods were chosen to approximate diel cycles characteristic of current and hypothetical future niches of *P*.*marinus* and delivered approximating a sinusoidal sun rise and fall; 16 h represents temperate (45°N) summer at the ocean surface; 12 h for equatorial (0°N) ocean surface or temperate (45°N) spring and fall ocean surface or temperate (45°N) summer at deeper ocean depths; 8 h for temperate (45°N) winter at the surface or at temperate (45°N) spring and fall at depth and equatorial (0°N) deep ocean depths; and 4 h for temperate (45°N) winter at deep ocean depths.

### Growth rate analysis

Data files (.csv) saved from the Multicultivator software were imported into R-Studio for data management [46], growth rate calculations, comparisons of model fits [54], and visualization. The chlorophyll proxy optical density (OD_680_—OD_720_; ΔOD) was used to determine the chlorophyll specific growth rate (μ, d^-1^) for each treatment combination. We first used a rolling mean from the R package *zoo* [55] to calculate the average ΔOD data over a 1-hour window to lower the influence of outlier points and remove data points collected during post stationary phase, when applicable. We used the Levenberg-Marquardt algorithm [56] modification of the non-linear least squares, using the R package *minpack*.*lm* [57], to fit a logistic equation (Eq (1)); where ΔOD_max_ is maximum ΔOD, ΔOD_min_ is minimum ΔOD, t is time duration over the growth trajectory.


$$\mu =\frac{\Delta OD_{max}\times \Delta OD_{min}\times exp^{\left(\mu \times t\right)}}{\Delta OD_{max}+(\Delta OD_{min}\times exp^{\left((\mu \times t)-1\right)})}$$ (1)

S2 Fig is an example of chlorophyll specific growth rate estimates fitted from the high resolution ΔOD measurements for each tube in a Multicultivator. The residuals of the logistic growth rate fit are shown. The imposed PAR (μmol photons m^-2^ s^-1^) are plotted for each tube and illustrates the applied photoperiod (h) regimes.

A Generalized Additive Model (GAM) [58] was applied to the relation of chlorophyll-specific μ d^-1^ to photoperiod and PAR level, for each growth [O_2_] level, and for the blue and red wavebands for growth, for each *P*. *marinus* strain in this study. The R package *mgcv* [59] was used to model the growth rate with smoothing terms and indicate the 90, 50 and 10% quantiles for growth rate across the levels of factors. Only growth rate estimates for which the amplitude of standard error was smaller than 30% of the fitted growth rate were included in the GAM. Our priority was the effects of ecologically relevant blue light on growth rate trends. We also included GAM analyses of growth rate responses to red light, which is not ecophysiologically relevant, but which might prove mechanistically informative [60].

### Estimation of photosynthetically usable radiation

To estimate the Photosynthetically Usable Radiation (PUR), a proxy of incident photons that can be absorbed by the cells, for each *P*. *marinus* ecotype, the imposed Photosynthetically Active Radiation (PAR) was first determined using the reported delivery of sinusoidal diel PAR regimes by the Multicultivators, point validated using a LI-250 quantum sensor (LI-COR Inc.,Lincoln, NE, USA). An emission profile from 400 nm to 700 nm of each coloured LED light of the MCMIX-OD Multicultivator and the white LED light of the MC1000-OD Multicultivator was obtained using a Jaz spectrometer (Ocean Optics, Inc.,Dunedin, FL, USA) equipped with a fiber optic cable, HH2 FiberOpticJmp (Part number A901073, Malvern Panalytical Ltd, Malvern, UK). Each LED spectrum was then normalized to its emission maximum. An *in-vivo* whole cell absorbance spectrum for each *P*. *marinus* strain under each spectral growth condition was obtained using the Olis 14 UV/VIS Clarity Spectrophotometer (Olis Inc., Bogart, GA, USA) to scan across range of λ = 350 nm to 750 nm at 1 nm intervals. The path length of the internally reflective cavity of the Olis spectrophotometer was corrected to a 1 cm path length using the Javorfi correction method [61] on PRO 99 media subtracted whole cell absorbance spectra. The blank-corrected whole cell absorbance spectra were normalized to the absorbance maximum of divinyl chlorophyll *a* (Chl *a*_2_), determined for each spectra, falling between 400 nm and 460 nm.

An integrated weighting Eq (2) [37] was used to determine the weighted PUR spectrum P(λ); where A(λ) is the blank subtracted, Chl *a*_2_ peak normalized whole cell absorbance spectrum for each *P*. *marinus* ecotype, over 400 nm to 700 nm, A(λ); and E(λ) is the peak normalized emission spectrum of the imposed LED growth light, over 400 nm to 700 nm.


P(λ)=A(λ)×E(λ) (2)

**Fig 1:**
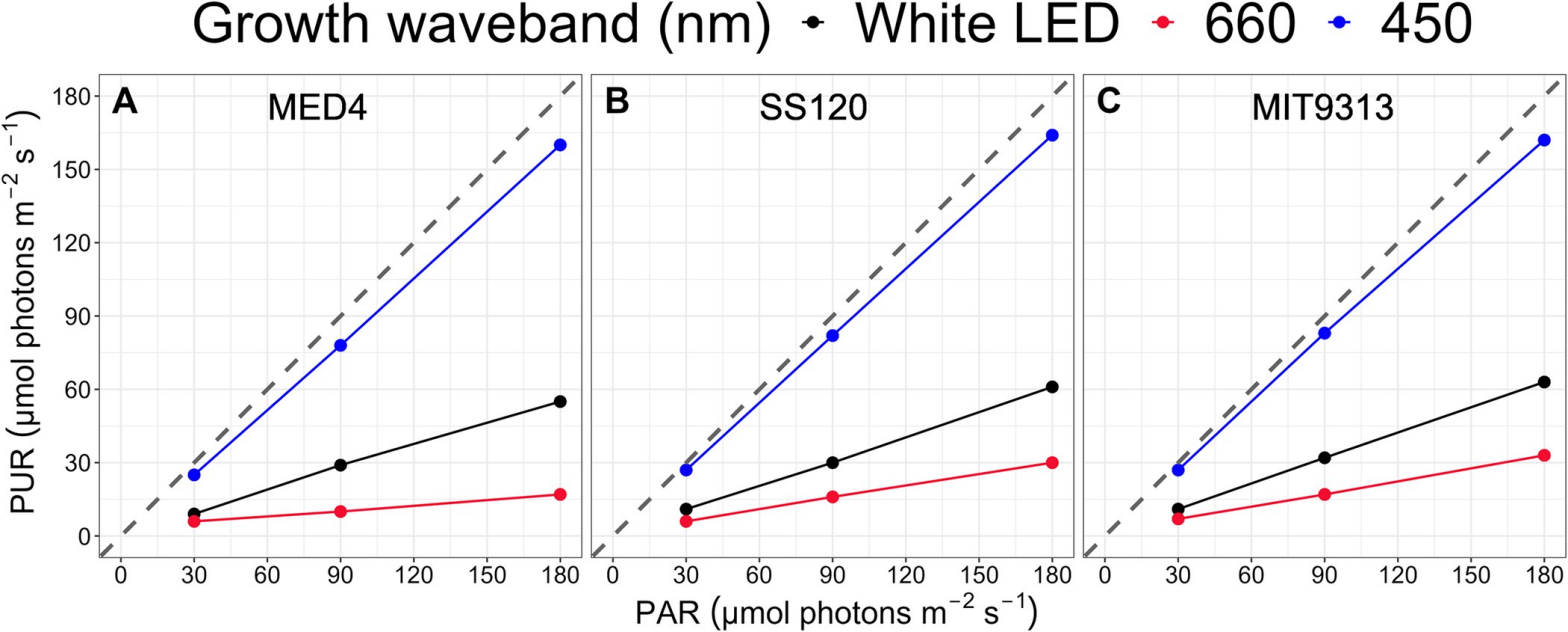
Peak Photosynthetically Usable Radiation (PUR; μmol photons m^-2^ s^-1^) vs. peak Photosynthetically Active Radiation (PAR; μmol photons m^-2^ s^-1^). The correlation between PAR, plotted on the x-axis and PUR, plotted on the y-axis, are coloured for each imposed spectral waveband; 450 nm (blue circles), 660 nm (red circles) and white LED (black circles). The grey dashed line represents a hypothetical one to one correlation. **A.** is *Prochlorococcus marinus* MED4. **B.** is *Prochlorococcus marinus* SS120. **C.** is *Prochlorococcus marinus* MIT9313.

PUR levels (μmol photons m^-2^ s^-1^) were calculated from imposed PAR (μmol photons m^-2^ s^-1^) levels using the Eq (3) from [37]; where P(λ) is the weighted PUR absorbance spectrum from Eq (2), E(λ) is the imposed growth light emission spectrum from Eq (2) and PAR is the imposed peak light level (μmol photons m^-2^ s^-1^). Fig 1 shows the calculated peak PUR (μmol photons m^-2^ d^-1^) vs. imposed peak PAR (μmol photons m^-2^ s^-1^) for each strain and each spectral waveband (nm).


$$PUR=\frac{\int_{400}^{700}P(\lambda )}{\int_{400}^{700}E(\lambda )}\times PAR$$ (3)

The applied photoperiods were delivered using the sinusoidal circadian light function of the PSI Multicultivator to simulate light exposure approximating sun rise through to sunset. The area under the sinusoidal curves is equivalent to the area of a triangular photoregime of equivalent photoperiod, therefore the equation to determine the cumulative diel PUR (μmol photons m^-2^ d^-1^) is one half of the base (photoperiod) multiplied by the height (PUR) (Eq (4)); where PUR is the usable light (μmol photons m^-2^ s^-1^) calculated from Eq (3), 3600 is the time conversion from seconds to hour and photoperiod is the imposed photoperiod (h).


$$Cumulative\;diel\;PUR=\frac{PUR\times 3600\times Photoperiod}{2}$$ (4)

S3 Fig provide visual representations of PUR, the black solid line and shaded area, in relation to the imposed PAR, the dotted line, under each imposed spectral wavebands for *P*. *marinus* MED4 (A-C), SS120 (D-F) and MIT9313 (G-I). Fig 1 shows the relationship between calculated PUR vs. imposed PAR for each *P*. *marinus* and each spectral waveband.

We performed one-way ANOVA to examine statistical differences between Harrison and Platt [54] 4 parameter model fit to 660 nm (red light) and 450 nm (blue light) growth rate data for each combination of strain and [O_2_]. We also performed one-way ANOVA to examine statistical differences between Harrison and Platt [54] 4 parameter model fit to each photoperiod (4 h, 8 h, 12 h, 16 h) and pooled photoperiod growth rate data for each combination of strain and [O_2_]. Photoperiod growth rate data that showed complete growth inhibition for each combination of strain, [O_2_] and imposed spectral waveband were omitted from the pooled photoperiod model. Statistical differences were determined at *P* value < 0.05.

### *Prochlorococcus marinus* comparative genomics

We filtered the dataset of Omar *et al*. [62], for Enzyme Commission Numbers (EC numbers), or Kegg Orthology Numbers (KO numbers) identified by BRENDA [41] as ‘natural substrates’ for O_2_; EC numbers identified by BRENDA as being activated, or inhibited by light; and EC numbers annotated by BioCyc [63] as corresponding to the Gene Ontology Term (GO:0006281—DNA repair), in *P*. *marinus* strains (MED4, NATL2A, SS120, and MIT9313). We grouped orthologs together by EC number and their KO number and determined the occurrences of individual orthologs encoding each EC number, or KO number when EC number was not available, in a given strain. We merged the dataset with a list of enzyme Michaelis constant (K_m_) values from other organisms, as K_m_ values from *P*. *marinus* were only available in the case of Ribulose bisphosphate carboxylase. Gene counts for Flavodiirons were obtained from Allahverdiyeva *et al*. [64], as they do not have allocated EC numbers. A full list of enzymes and corresponding EC and KO numbers can be found in S1 Table.

## Results and discussion

### Detection of proteins from *Prochlorococcus marinus* Clades across O_2_ and light niches in the ocean

**Fig 2:**
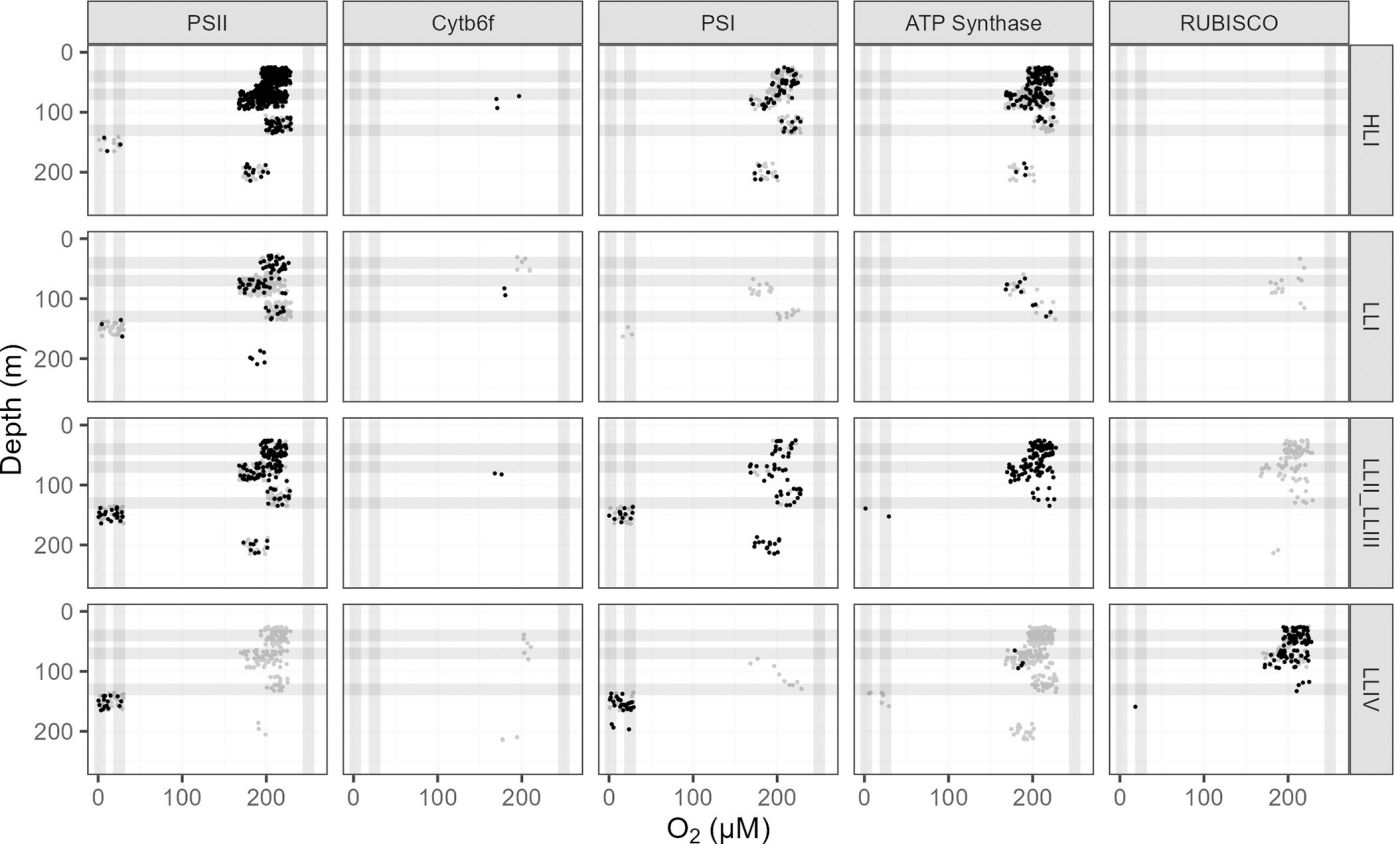
Ocean detection of *Prochlorococcus marinus* photosynthesis complexes. Protein detections (circles) are plotted vs. O_2_ (μM) (x-axis) and depth (m) (y-axis) at sample origins, with ‘jitter’ offsets up to 15% of full axes scales, to visualize over-laid data points. Rows separate data annotated as from *Prochlorococcus marinus* clades: HLI (including *P*. *marinus* MED4, solid black circles), LLI (including *P*. *marinus* NATL2A, solid black circles), LLII/III (including *P*. *marinus* SS120, solid black circles) and LLIV (including *P*. *marinus* MIT9313, solid black circles). Columns show detections of proteins annotated as Photosystem II (PSII), Cytochromeb6f complex (Cytb6f), Photosystem I (PSI), ATP Synthase or Ribulose-1,5-bisphosphate oxygenase carboxylase (RUBISCO). For comparison, experimental conditions for culture growth rate determinations are indicated by horizontal grey lines for depths, approximating peak Photosynthetically Active Radiation (PAR; μmol photons m^-2^ s^-1^); and vertical grey lines for imposed [O_2_] (μM). Data obtained from the Biological and Chemical Oceanography Data Management Office repository [43].

Proteins from *P*. *marinus* were detected across depths and oxygen concentrations in the OPP data set [43]. We focused our analyses on core photosynthetic protein complexes, annotated as coming from clades HLI (2 strains, including strain MED4); LLI (2 strains, including strain NATL2A); LLII/III (2 strains, including strain SS120) and LLIV (2 strains, including MIT9313) (Fig 2), as a function of depth (a proxy for light level) and measured [O_2_] at the sampling locations. Photosynthetic complexes from clades HLI and LLI were detected throughout the water column, although predominately at stations with high [O_2_]. Though present and expressed in the genome of MED4 [65], the absence of proteins annotated as RUBISCO complex for clade HLI, compared to annotated detections of RUBISCO across the other three clades, suggests limitations in the annotation process assigning highly conserved protein sequences to clades. Notwithstanding limitations on assignments of proteins to clades, detections of the abundant carbon fixation complex RUBISCO derived from clades HLI, LLI and LLII/III were notably absent from stations at low [O_2_], suggesting limited capacity for carbon fixation by clades HLI, LLI and LLII/LLIII under low [O_2_] habitats. Clade LLII/LLIII photosynthetic complexes were detected throughout the sampled ranges of depth/light and [O_2_], with detections of proteins at both high and low [O_2_], compared to other strains. Complexes from LLIV (including strain MIT9313) were detected across the depth/light and [O_2_] ranges, with more detections at deeper, darker depths and at low [O_2_], compared to other clades. We note that detections of photosynthetic proteins from strain MIT9313 (dark symbols) show a particular bias towards low [O_2_] sampling stations, compared to the wider detections of clade LLIV. Our analyses utilized a proteomics dataset; however, alternate approaches, such as metagenomics or metatranscriptomics, could have been employed to analyze ecotype abundances using the TARA Oceans [66] or other datasets.

### *Prochlorococcus marinus* growth rate responses to photoperiod, PAR, spectral band, and [O_2_]

Guided in part by the evidence of ocean distributions of proteins from *P*. *marinus*, we set up a matrix of photoperiods, PAR, and [O_2_] to approximate current, and potential future, latitudinal, depth and seasonal niches for *P*. *marinus* strains. As mentioned, growth rate trials under red light, although not representative of *P*. *marinus* niches, are mechanistically informative [60] regarding photoinactivation of PSII. We implemented measures to minimize shock to cultures from exposure to experimental growth conditions by inoculating them the day before the experiment began, employing a sinusoidal photoperiod with a gradual increase in PAR exposure, and extracting growth rates from logistic curves fit over approximately 5 generations of growth, to accommodate multiple generations to acclimate to the imposed growth conditions [6]. Although the current subtropical distribution of *P*. *marinus* spans a narrow range of photoperiods at the surface, light attenuation with depth shortens effective photoperiods as well as lowering peak PAR. Potential poleward latitudinal range expansions, in combination with attenuation of light with depth, mean *P*. *marinus* clades may potentially encounter a wider range of photoperiods. Our growth rate determinations generally agree with those from Moore *et al*. [5], for white LED and 250 μM O_2_, but our study is, to our knowledge, the first to analyze the interactive growth rate responses of *P*. *marinus* strains to varying [O_2_], photoperiods and spectral wavebands.

**Fig 3:**
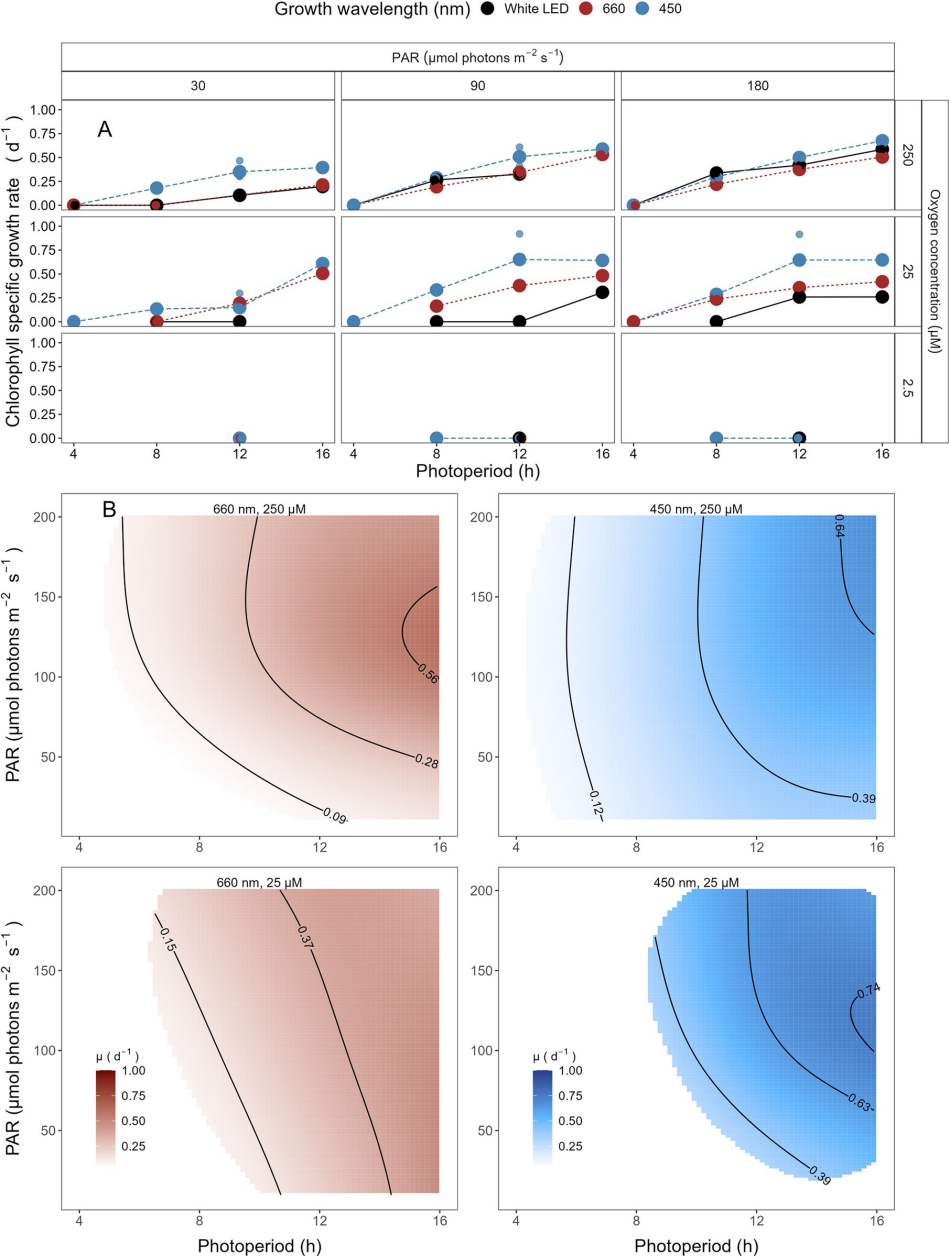
**A. Chlorophyll specific growth rate (d**^**-1**^**) for *Prochlorococcus marinus* MED4 (High-Light (HLI) near surface clade) vs. photoperiod (h).** Rows separate data from levels of imposed dissolved O_2_ concentrations (250 μM, 25 μM and 2.5 μM). Columns separate data from 3 levels of peak imposed Photosynthetically Active Radiation (PAR; 30, 90 and 180 μmol photons m^-2^ s^-1^). Colours represent the imposed spectral waveband (nm). Large circles show mean or single determinations of growth rate from logistic curve fits; small circles show values for replicate determinations, if any: Replicates often fall with larger circles. **B. Contour plot of a Generalized Additive Model (GAM) representing the chlorophyll specific growth rate (d**^**-1**^**) for *Prochlorococcus marinus* MED4 grown under 660 nm (red) or 450 nm (blue) light.** X-axis is photoperiod (h). Y-axis is Photosynethetically Active Radiation (PAR, μmol photons m^-2^ s^-1^). Top left panel represents the model under 250 μM of O_2_ and red light. Bottom left panel represents the model under 25 μM of O_2_ and red light. Top right panel represents the model under 250 μM of O_2_ and blue light. Bottom right panel represents the model under 25 μM of O_2_ and blue light. Legends represent a colour gradient of growth rate from no growth (white) to 1.00 d^-1^ (dark red or dark blue). Labeled contour lines indicate the 90%, 50%, and 10% quantiles for achieved growth rate.

The growth rate for *P*. *marinus* MED4, clade HLI, under 250 μM O_2_, increased with higher imposed PAR and longer photoperiods (Fig 3A), across all spectral wavebands. No growth was observed under any imposed conditions under a 4 h photoperiod. The maximum growth rate (μ_max_) was 0.68 d^-1^ achieved under 180 μE blue light and 16 h photoperiod.

Similar to growth rate trends under 250 μM O_2_, MED4 maintained at 25 μM O_2_ showed no growth under any imposed conditions under a 4 h photoperiod and the growth rate increased with higher imposed PAR and longer photoperiods (Fig 3A). The μ_max_ was 0.65 d^-1^ (S2 Table) achieved under 90 μmol photons m^-2^ s^-1^ blue light and 12 h photoperiod. The 4 h photoperiod experiments under white LED light were not performed as no growth was achieved when grown under an 8 h photoperiod of white LED light.

MED4 did not grow when sparged to the lowest [O_2_] of 2.5 μM (Fig 3A). 2.5 μM O_2_ growth rate experiments were not conducted for 4 and 16 h photoperiods, as no reproducible growth occurred when MED4 was exposed to 8 and 12 h photoperiods under [O_2_] of 2.5 μM.

The GAM model in Fig 3B summarizes MED4 growth rate responses to red or blue peak PAR and photoperiod across 2 imposed oxygen concentrations. Under 250 μM O_2_ MED4 achieved fastest growth rates above peak blue light of ~180 μmol photons m^-2^ s^-1^, and the longest photoperiod of 16 h, indicated by the 0.64 d^-1^ contour line representing the 90^th^ percentile of maximum achieved growth rate (Fig 3B). Growth rates decreased with decreasing photoperiod and decreasing peak PAR. Under red light, growth rates were generally slower but the pattern of growth rate responses to photoperiod and PAR was similar (Fig 3B). Note the exclusion of MED4 from growth under 4 h photoperiod under both red and blue light (Fig 3B). Under 25 μM O_2_ MED4 showed similar growth rate responses, but was excluded from both 4 and 8 h photoperiods. MED4 did not grow under 2.5 μM O_2_, so no GAM model was run. Considering the range of PAR levels, and spectral wavebands that MED4 can utilize, MED4 can inhabit not just shallow depths, where light levels are high, but also deeper regions, characterized by a lower level of blue light, subject to the limitation of a photoperiod of more than 4 h, even after depth attenuation of light. The photoregimes of winter temperate zones, due to shorter photoperiods, exclude MED4 from growth at deeper regions, however temperate photoperiods and light levels for the remainder of the year are potentially adequate to support MED4 growth, if water temperatures warm into the clade HLI tolerance range.

**Fig 4:**
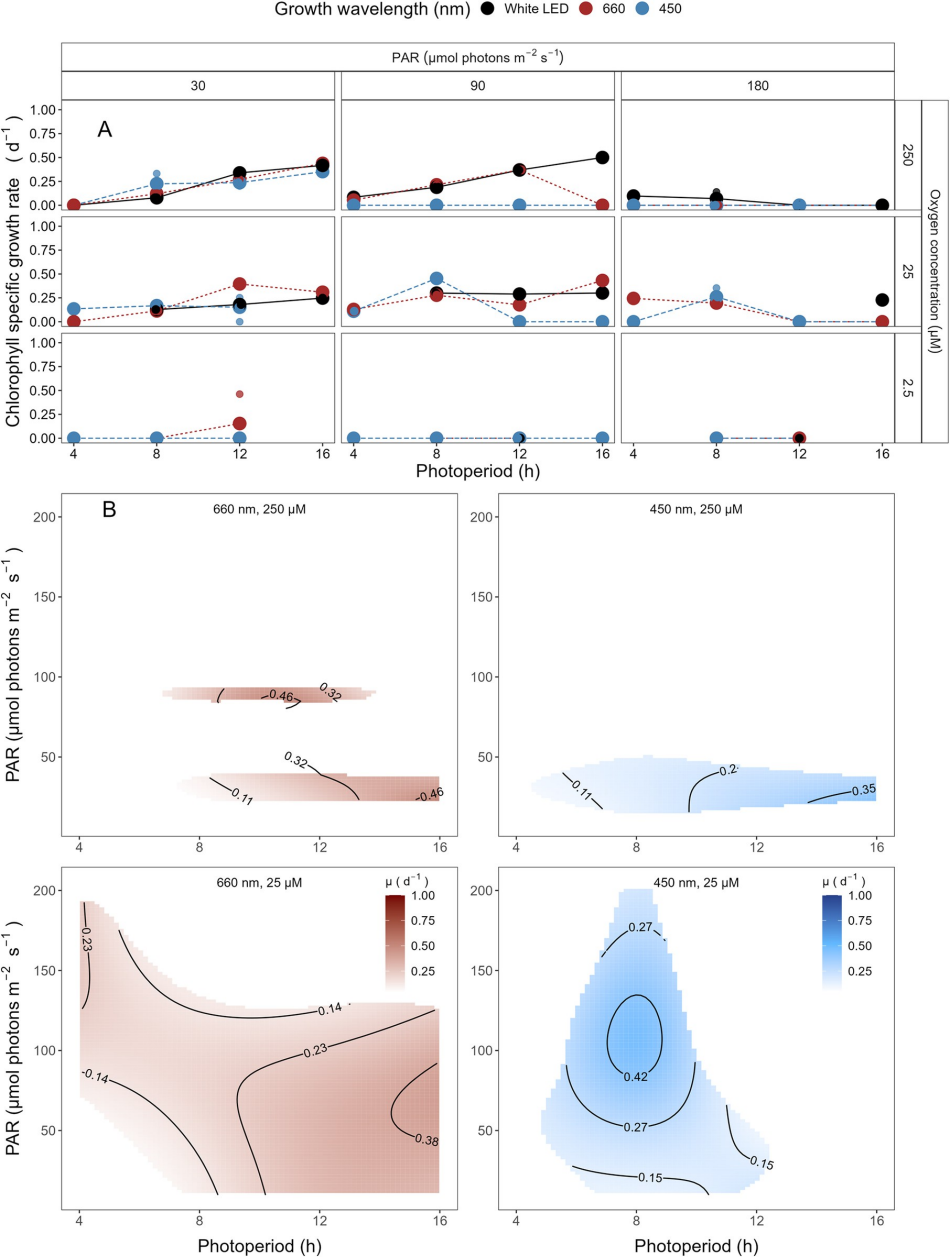
**A. Chlorophyll specific growth rate (d**^**-1**^**) for *Prochlorococcus marinus* SS120 (Low-Light, deep ocean clade LLII/III) vs. photoperiod (h).** Rows separate data from levels of imposed dissolved O_2_ concentrations (250 μM, 25 μM and 2.5 μM). Columns separate data from 3 levels of peak imposed Photosynthetically Active Radiation (PAR; 30, 90 and 180 μmol photons m^-2^ s^-1^). Colours represent the imposed spectral waveband (nm). Large circles show mean or single determinations of growth rate from logistic curve fits; small circles show values for replicate determinations, if any: Replicates often fall with larger circles. **B. Contour plot of a Generalized Additive Model (GAM) representing the chlorophyll specific growth rate (d**^**-1**^**) for *Prochlorococcus marinus* SS120 grown under 660 nm (red) or 450 nm (blue) light.** X-axis is photoperiod (h). Y-axis is Photosynethetically Active Radiation (PAR, μmol photons m^-2^ s^-1^). Top left panel represents the model under 250 μM of O_2_ and red light. Bottom left panel represents the model under 25 μM of O_2_ and red light. Top right panel represents the model under 250 μM of O_2_ and blue light. Bottom right panel represents the model under 25 μM of O_2_ and blue light. Legends represent a colour gradient of growth rate from no growth (white) to 1.00 d^-1^ (dark red or dark blue). Labeled contour lines indicate the 90%, 50%, and 10% quantiles for achieved growth rate.

The growth rates for *Prochlorococcus marinus* SS120 clade LLII/III, under 250 μM O_2_, 30 μmol photons m^-2^ s^-1^ peak PAR and across all spectral wavebands, increased with longer photoperiods (Fig 4A). No growth was observed under any blue light photoperiods when exposed to peak PAR of 90 μmol photons m^-2^ s^-1^ or greater. Growth rate, however increased with increasing photoperiods for white and red light under peak PAR of 90 μmol photons m^-2^ s^-1^ but showed growth inhibition at 16 h red light photoperiod. Growth rate decreased with longer photoperiods and showed growth inhibition at 12 and 16 h photoperiods under PAR of 180 μmol photons m^-2^ s^-1^ white LED and growth inhibition across all photoperiods under red or blue light. The μ_max_ was 0.5 d^-1^ (S2 Table) achieved under 90 μmol photons m^-2^ s^-1^ white LED light and 16 h photoperiod.

Under 25 μM O_2_ and PAR of 30 μmol photons m^-2^ s^-1^ growth rate trends were similar to 250 μM O_2_. SS120 showed no growth under a 4 h photoperiod for red spectral waveband, however under blue light, SS120 was able to grow (Fig 4A). In contrast to the growth rate trends of the 250 μM O_2_ and PAR of 90 μmol photons m^-2^ s^-1^ experiments, SS120 grew under 4 and 8 h blue light and 16 h red light photoperiods, however the growth rate decreased under 12 and 16 h white LED light photoperiod treatments. Blue light treatments under PAR of 180 μmol photons m^-2^ s^-1^ showed growth only under an 8 h photoperiod. The μ_max_ was 0.45 d^-1^ (S2 Table) achieved under 90 μmol photons m^-2^ s^-1^ blue light and 8 h photoperiod. The 25 μM O_2_, less than 16 h photoperiod and 180 μmol photons m^-2^ s^-1^ under white LED light experiments were not performed due to time constraints.

SS120 did not reproducibly grow when sparged to the lowest O_2_ of 2.5 μM (Fig 4A). 2.5 μM O_2_ growth rate experiments were not conducted for 4 and 16 h photoperiods under peak PAR of 180 μmol photons m^-2^ s^-1^ and for red light under peak PAR of 90 μmol photons m^-2^ s^-1^, as no growth occurred when SS120 was exposed to 8 and 12 h photoperiods.

The GAM model in Fig 4B summarizes growth rate responses of SS120 to red or blue peak PAR and photoperiod, across the 2 imposed oxygen concentrations. Under 250 μM O_2_, Fig 4B showed highest growth rates below blue light PAR of 50 μmol photons m^-2^ s^-1^ and photoperiods between 12 and 16 h, indicated by the contour line labeled 0.35 d^-1^ (representing the 90^th^ percentile of achieved growth rate). Under 250 μM O_2_ SS120 is constrained to deeper ocean waters through its intolerance of higher blue PAR levels. These findings align with Moore *et al*. [5] and are expected for a low light clade. Growth rate patterns under red light and 250 μM O_2_ were similar, although somewhat faster. The disjunct regions of the GAM plot results from variable growth success of SS120 under 250 μM O_2_. In contrast, under 25 μM O_2_ and a photoperiod of 8 h SS120 exploited all blue peak PAR levels, achieving faster growth rates at a higher PAR of ~100 μmol photons m^-2^ s^-1^, indicated by the contour line labeled 0.42 d^-1^ (representing the 90^th^ percentile of achieved growth rate), out pacing the 90^th^ percentile fastest growth rates under 250 μM O_2_ (Fig 4B). Under red light and 25 μM O_2_ (Fig 4B) SS120 was able to grow across most conditions of peak PAR and photoperiod, achieving fastest growth rate under long photoperiods and peak PAR between 30 ~100 μmol photons m^-2^ s^-1^. Thus, the designation of SS120 as a LL strain is dependent upon the [O_2_]. SS120 did not, however, grow reliably under tested conditions at 2.5 μM O_2_.

**Fig 5:**
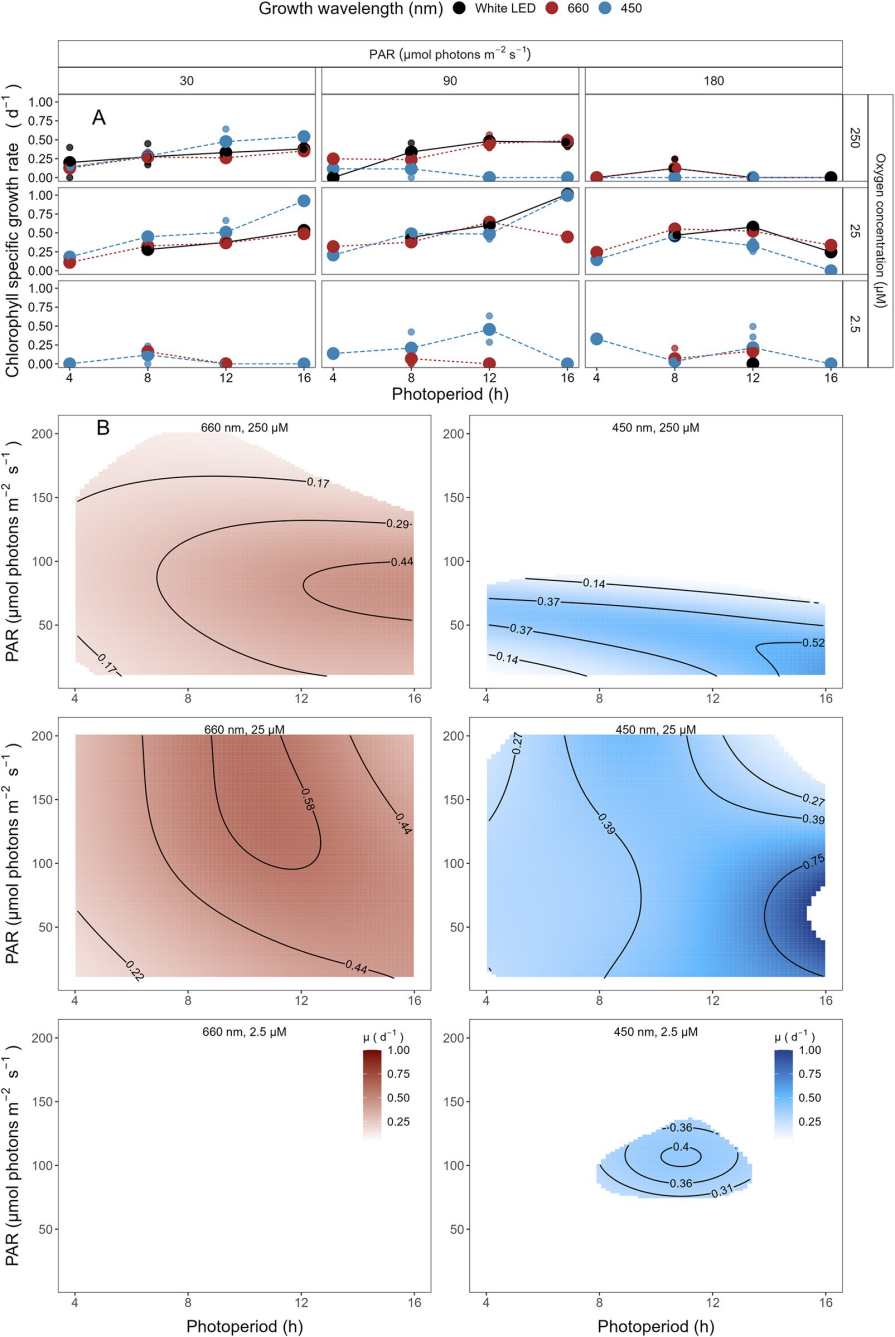
**A. Chlorophyll specific growth rate (d**^**-1**^**) for *Prochlorococcus marinus* MIT9313 (Low-Light, deep ocean clade LLIV) vs. photoperiod (h).** Rows separate data from levels of imposed dissolved O_2_ concentrations (250 μM, 25 μM and 2.5 μM). Columns separate data from 3 levels of peak imposed Photosynthetically Active Radiation (PAR; 30, 90 and 180 μmol photons m^-2^ s^-1^). Colours represent the imposed spectral waveband (nm). Large circles show mean or single determinations of growth rate from logistic curve fits; small circles show values for replicate determinations, if any: Replicates often fall with larger circles. **B. Contour plot of a Generalized Additive Model (GAM) representing the chlorophyll specific growth rate (d**^**-1**^**) for *Prochlorococcus marinus* MIT9313 grown under 660 nm (red) or 450 nm (blue) light.** X-axis is photoperiod (h). Y-axis is Photosynthetically Active Radiation (PAR; μmol photons m^-2^ s^-1^). Top left panel represents the model under 250 μM of O_2_ and red light. Center left panel represents the model under 25 μM of O_2_ and red light. Bottom left panel represents the model under 2.5 μM of O_2_ and red light. Top right panel represents the model under 250 μM of O_2_ and blue light. Center right panel represents the model under 25 μM of O_2_ and blue light. Bottom right panel represents the model under 2.5 μM of O_2_ and blue light. Legends represent a colour gradient of growth rate from no growth (white) to 1.00 d^-1^ (dark red or dark blue). Labeled contour lines indicate the 90%, 50%, and 10% quantiles for achieved growth rate.

*Prochlorococcus marinus* MIT9313, clade LLIV, growth rates under 250 μM O_2_ increased with longer photoperiods, under low 30 μmol photons m^-2^ s^-1^ peak PAR, (Fig 5A). Under intermediate 90 μmol photons m^-2^ s^-1^ peak PAR growth rates decreased with increasing blue light photoperiods. Blue light did not induce growth at 180 μmol photons m^-2^ s^-1^ peak PAR, while MIT9313 showed only marginal growth under white LED and red light at 180 μmol photons m^-2^ s^-1^ peak PAR, under the 8 h photoperiod, consistent with Moore *et al*. [35]. The μ_max_ was 0.54 d^-1^ achieved under 30 μmol photons m^-2^ s^-1^ blue light and 16 h photoperiod.

For MIT9313 under 25 μM O_2_, growth rate increased with increasing photoperiods for all spectral wavebands tested (Fig 5A), with the fastest overall growth rate for MIT9313 1.01 d^-1^ achieved under peak PAR of 90 μmol photons m^-2^ s^-1^ and 16 h white LED light photoperiod. In marked contrast to the 250 μM O_2_ growth rate experiments, MIT9313 grew when exposed to peak PAR of 180 μmol photons m^-2^ s^-1^ and blue light under all photoperiods except 16 h; additionally, white LED and red light treatments induced growth across all tested photoperiods under 25 μM O_2_.

MIT9313 grew under 2.5 μM O_2_ particularly under blue LED light, albeit generally slower than under the parallel experiments at 25 μM O_2_ (Fig 5A). Growth rates showed scatter among replicates, suggesting 2.5 μM O_2_ is near the tolerance limit for growth of MIT9313. Growth rates increased with longer photoperiods under blue light treatments and peak PAR of 90 μmol photons m^-2^ s^-1^ but did not grow under 16 h photoperiod. Growth rates for MIT9313 under PAR of 180 μmol photons m^-2^ s^-1^ and blue light treatment decreased with increasing photoperiods with full growth inhibition under a 16 h photoperiod. The red light peak PAR of 180 μmol photons m^-2^ s^-1^ showed similar growth rates to blue light for 8 and 12 h photoperiods. The μ_max_ was 0.45 d^-1^, achieved under 12 h blue light photoperiod and PAR of 90 μmol photons m^-2^ s^-1^. The 2.5 μM O_2_ white LED treatments under 4, 8 and 16 h photoperiods and red light under 4 and 16 h photoperiods were not performed as cultures were unlikely to grow.

The GAM model in Fig 5B summarizes MIT9313 growth rate responses to red or blue peak PAR and photoperiod. Under 250 μM O_2_, Fig 5B shows MIT9313 achieves fastest growth rates between blue peak PAR of 30 μmol photons m^-2^ s^-1^ and 50 μmol photons m^-2^ s^-1^ and photoperiods longer than 12 h, indicated by the contour line labeled 0.52 d^-1^ representing the 90^th^ percentile of achieved growth rates. Fig 5B also shows that growth rate increases with longer photoperiods, as long as the blue peak PAR levels remain below 50 μmol photons m^-2^ s^-1^. In contrast, under red light and 250 μM O_2_ MIT9313 is able to exploit higher peak PAR, across the range of photoperiods. MIT9313 can exploit all blue or red PAR levels and most photoperiods under 25 μM O_2_ with 90^th^ percentile of fastest growth rate between 30 to 100 μmol photons m^-2^ s^-1^ blue PAR. MIT9313 maintains growth even under 2.5 μM O_2_, under photoperiods between 8 and 12 h and peak blue PAR of ~100 μmol photons m^-2^ s^-1^. Thus the designation of MIT9313 as a LL clade is dependent upon [O_2_] and light spectra (Fig 5B).

### PUR and growth rate responses

Cumulative diel PUR can potentially collapse photoperiod, PAR and spectral wavebands to a common metric of usable photosynthetically active light per day. Cumulative diel PUR (μmol photons m^-2^ d^-1^) was calculated from PUR (μmol photons m^-2^ s^-1^) and photoperiod (h) (Eq (4)). We plotted growth rates vs. cumulative diel PUR to determine whether growth rate is a simple response to diel PUR, across imposed photoperiods and spectral wavebands, or whether photoperiods or spectral wavebands have specific or interactive influences on growth, beyond cumulative diel PUR.

Due to the absorption of *P*. *marinus* pigments in the blue spectral waveband range, the maximum cumulative diel PUR under blue light is almost 3 times that of white LED light, and about 5 times that of the red light (Fig 1), despite being derived from the same photoperiods and peak PAR regimes. As such, only blue light experiments extend beyond a cumulative diel PUR of ~2 x 10^6^ μmol photons m^-2^ d^-1^. This spectral bias in the range of PUR leads us to caution in comparing model fits of growth rate in response to cumulative diel PUR under red vs. blue wavebands. Furthermore, we found some distinct model fits for specific photoperiods, contributing to scatter within the red vs. blue data sets.

**Fig 6:**
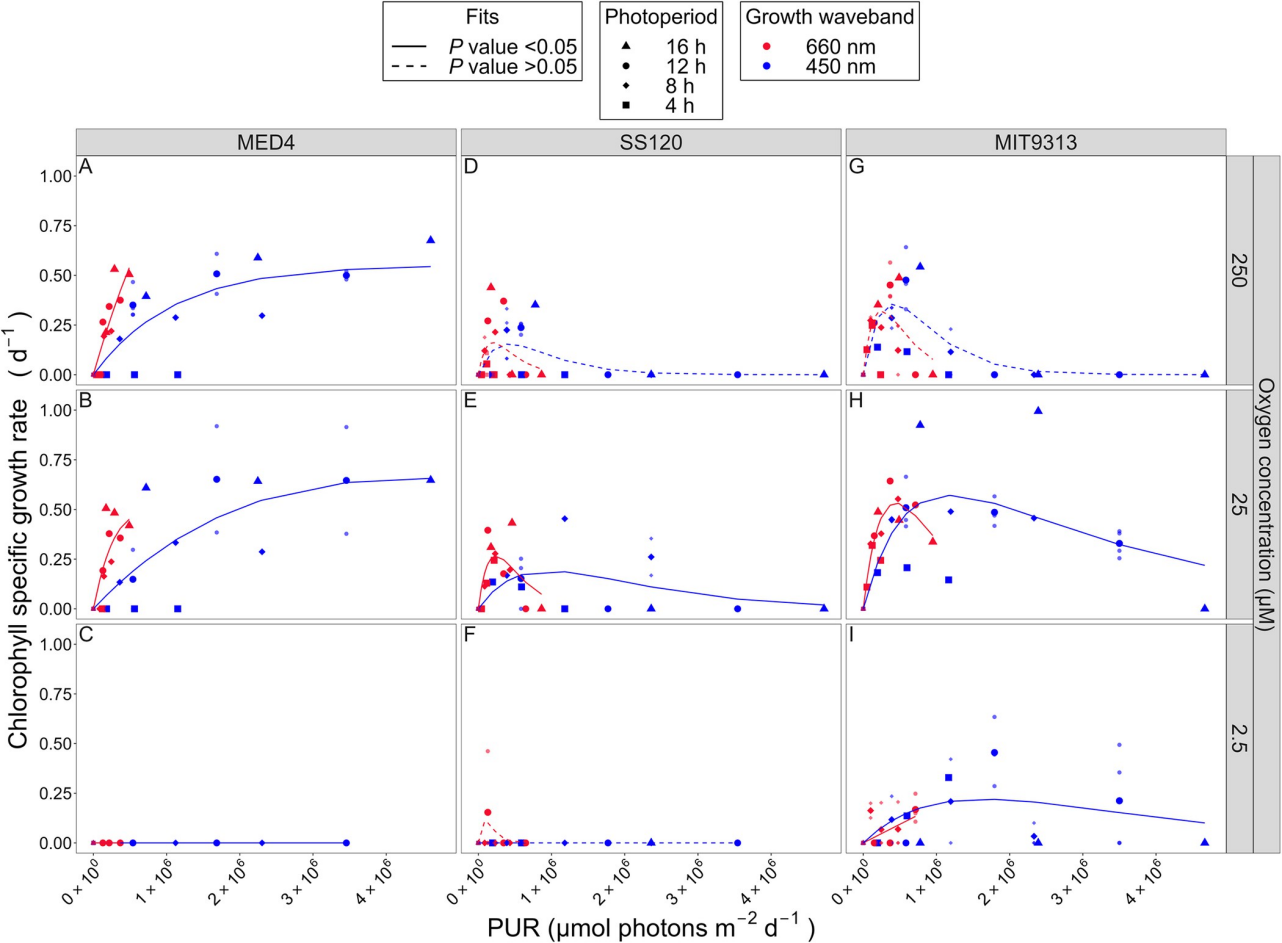
Chlorophyll specific growth rate (d^-1^) vs. cumulative diel Photosynthetically Usable Radiation (PUR, μmol photons m^-2^ d^-1^). Rows separate data from levels of imposed dissolved O_2_ concentrations as 250 μM, 25 μM and 2.5 μM. Columns separate data from strains; MED4 (A-C), SS120 (D-F) and MIT9313 (G-I). Shapes show the imposed photoperiod (h); 4 h (solid square), 8 h (solid diamond), 12 h (solid circle), 16 h (solid upright triangle). Symbol colours show the spectral waveband for growth; 660 nm (red symbols), and 450 nm (blue symbols). Large symbols show mean of growth rate from logistic curve fits; small symbols show values from replicates, if any. Harrison and Platt [54] 4 parameter model fit to 660 nm (red lines) or 450 nm (blue lines) growth rate data for each combination of strain and dissolved oxygen shown with solid lines (red significantly different from blue, *P* value < 0.05) or dashed lines (red not significantly different from blue, *P* value > 0.05) tested using one-way ANOVA comparisons of fits.

The representative of clade HLI, *P*. *marinus* MED4, showed no growth under any 4 h photoperiod treatments, even when a 4 h photoperiod delivered cumulative diel PUR equivalent to other photoperiod treatments (S4A–S4C Fig). In parallel MED4 showed no growth under 2.5 μM O_2_, no matter the level of diel cumulative PUR. In contrast, under 250 or 25 μM O_2_, and including photoperiods greater than 4 h, MED4 growth under blue light was described by a saturating response of growth rate [54] to increasing cumulative diel PUR, with saturation of growth rate achieved around 3.0 x 10^6^ μmol m^-2^d^-1^ (Fig 6A and 6B), and no evidence of inhibition of growth at any achieved cumulative diel PUR. Under the ‘artificial’ growth treatment of red light, MED4 achieved more growth per unit diel cumulative PUR (Fig 6A and 6B), consistent with Murphy *et al*. [60], who showed a lower cost for growth under red light, for MED4, because red light provokes less photoinactivation of PSII, than equivalent levels of blue light. For distinct fits for different photoperiods refer to S4A–S4C Fig.

The representative of clade LLII/III, *P*. *marinus* SS120 showed almost no growth under 2.5 μM O_2_ experiments (S4F Fig). Most 4 h photoperiod treatments of SS120 also did not grow under 250 μM O_2_, even when a 4 h photoperiod delivered cumulative diel PUR equivalent to other photoperiod treatments (S4D Fig). Again using [54], SS120 did not grow when exposed to more than ~1.0 x 10^6^ μmol photons m^-2^ d^-1^ of cumulative diel PUR under any spectral waveband or photoperiod combination, under 250 μM O_2_ (S4D Fig).

Under both 25 and 250 μM O_2_ experiments, SS120 growth rate plateaued by about 5.0 x 10^5^ μmol photons m^-2^ d^-1^ diel PUR, with some scatter among photoperiod and spectral waveband regimes. The onset of growth inhibition extended to higher cumulative diel PUR for cultures under 25 μM O_2_, showing that SS120 is partially protected from photoinhibition of growth by 25 μM O_2_. Under 25 μM O_2_, red light again generated more growth of SS120 per unit cumulative diel PUR, than did blue light, consistent with lower cost of growth through lower photoinactivation under red light (Fig 6E) [60]. For distinct fits for different photoperiods refer to S4D–S4F Fig.

The clade LLIV representative, *P*. *marinus* MIT9313, under 250 μM O_2_ showed growth rate rising to a plateau by about 5 x 10^5^ μmol photons m^-2^ d^-1^ of cumulative diel PUR. Above about 1.0 x 10^6^ μmol photons m^-2^ d^-1^ of cumulative PUR under 250 μM O_2_, MIT9313 showed full inhibition of growth, across photoperiods, and spectral wavebands (S4G Fig). Under 25 μM O_2_ MIT9313 showed higher growth rates over a wider plateau, with a greatly extended exploitation of higher cumulative diel PUR, with full growth inhibition only above about 3.5 x 10^6^ μmol photons m^-2^ d^-1^ (S4H Fig). Under 2.5 μM O_2_, MIT9313 showed a wider, lower, flatter growth rate response to cumulative diel PUR, with full growth inhibition only above about 3.5 x 10^6^ μmol photons m^-2^ d^-1^ cumulative diel PUR (S4I Fig).

As with MED4 and SS120, our data again support enhanced growth rates under conditions of low cumulative diel PUR and 660 nm (red) spectral bandwidth, consistent with Murphy *et al*. [60] who found a lower cost of growth, due to decreased photoinactivation of PSII under red, compared to blue, wavebands (Fig 6G and 6H). Interestingly, this protective effect of red light disappears for MIT9313 growing under 2.5 μM O_2_, possibly because photoinactivation is strongly suppressed under this low [O_2_] (Fig 6I). For distinct fits for different photoperiods refer to S4G–S4I Fig.

### Photosystem II maintenance, oxygen metabolism, and DNA repair as limitations on *Prochlorococcus marinus* growth

Under full atmospheric [O_2_] and blue light, LL clades of *P*. *marinus* are restricted to growth under low light, in part because they suffer photoinhibition of Photosystem II (PSII) through several paths, including direct absorbance of UV or blue light, in parallel with generation of ROS if the electron flow is slowed [67], producing damaging singlet oxygen (^1^O_2_) [60,67–69]. Repair of photoinactivated PSII relies on the removal of damaged PsbA [70,71], followed by reassembly with newly synthesized PsbA [72]. Degradation of PsbA is a rate-limiting step in recovery from photoinhibition [73], mediated largely by a heterohexamer, termed in *P*. *marinus* (FtsH1-FtsH2)_3_, a membrane-bound metalloprotease [74–76].

*Prochlorococcus marinus* genomes encode 4 FtsH proteins [70,77], henceforth referred to as FtsH1-4, homologs to the characterized FtsH isoforms of the model freshwater cyanobacterium *Synechocystis sp*. PCC6803, and with presumably parallel functions (Table 1). Upon a shift to higher light, clade HLI MED4 upregulates expression of FtsH1 and FtsH2 [48], homologs to the *Synechocystis slr*0228 and *slr*1604, implicated in PSII repair [75,77]. In contrast, representative clade LLIV strain MIT9313 shows no induction of expression of these FtsH protease isoforms when shifted to high light, and thus has fewer of these FtsH hexamers serving each photosystem [48]. Transcript analysis demonstrates that MIT9313 expressed primarily FtsH3, homologous to *Synechocystis sll*1463, possibly involved in PSI biogenesis [70,78,79]. FtsH3 expression did not increase in response to light stress in MIT9313 [48]. Through adaptation to steady low light, clade LLIV *P*. *marinus* instead allocates resources to processes other than dynamic regulation of PSII repair.

**Table 1:** FtsH protease homologs in *Prochlorococcus marinus* and the model cyanobacterium *Synechocystis* sp. PCC6803. Protein homologies for FtsH isoforms between *Prochlorococcus marinus* and *Synechocystis* were extracted from [48].

Organism	Homolog 1	Homolog 2	Homolog 3	Homolog 4
*Prochlorococcus marinus*	FtsH1	FtsH2	FtsH3	FtsH4
*Synechocystis sp*. PCC6803	*Slr*0228	*Slr*1604	*Sll*1463	*Slr*1390
*Synechocystis sp*. PCC6803 isoform	FtsH2	FtsH3	FtsH4	FtsH1
Function	PSII Repair	PSII Repair	PSI biogenesis	Cell viability

**Fig 7:**
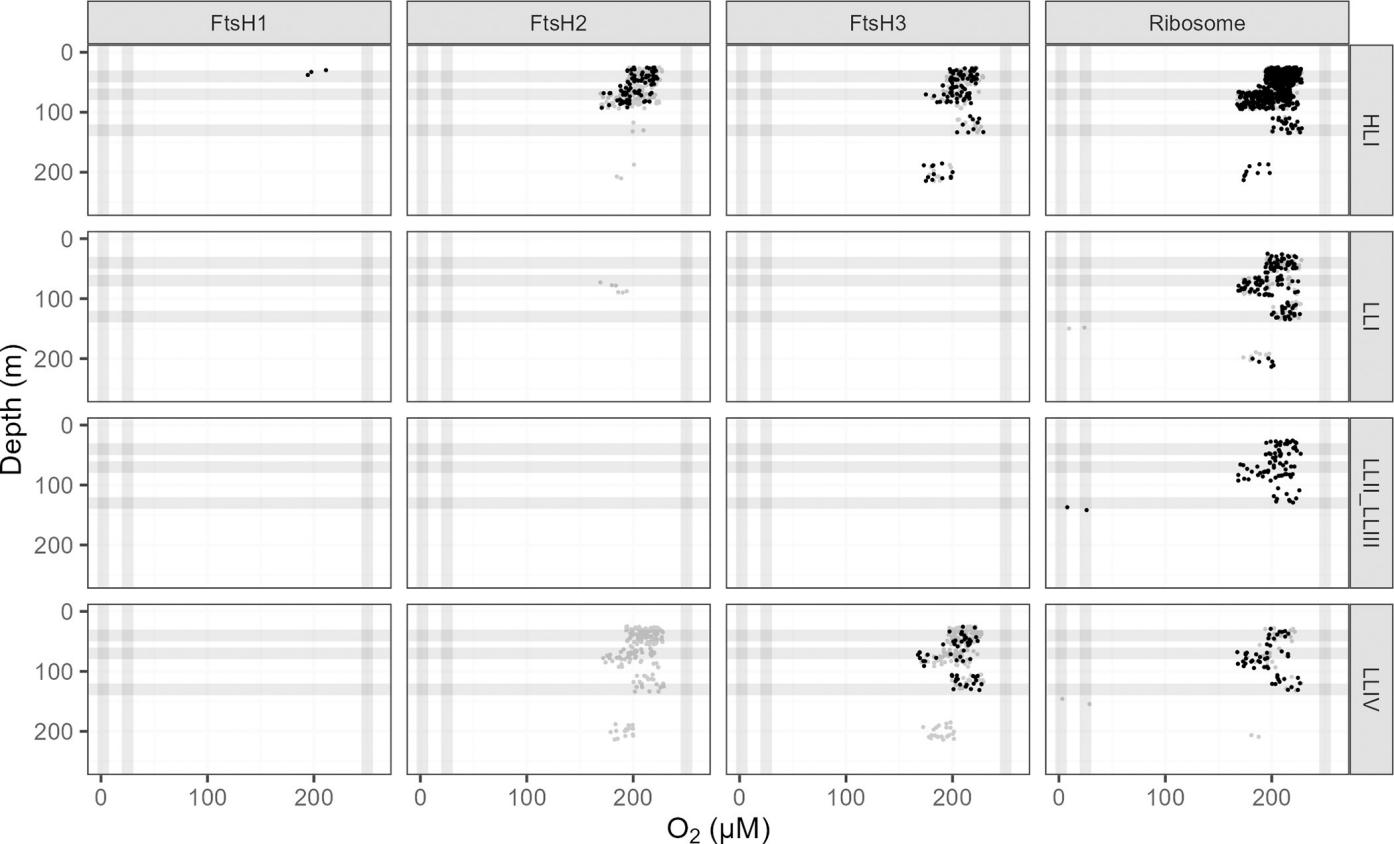
Ocean detection of *Prochlorococcus marinus* protein metabolism complexes. Protein detections (circles) are plotted vs. O_2_ (μM) (x-axis) and depth (m) (y-axis) at sample origin with a 15% offset to separate protein detections occupying the same origin. Rows separate data annotated as from *Prochlorococcus marinus* clades: HLI (including *P*. *marinus* MED4, solid black circles), LLI (including *P*. *marinus* NATL2A, solid black circles), LLII/III (including *P*. *marinus* SS120, solid black circles) and LLIV (including *P*. *marinus* MIT9313, solid black circles). Columns show detections of proteins annotated as FtsH Protease Complexes (FtsH1, FtsH2, FtsH3) or the Ribosome. For comparison, experimental conditions for culture growth rate determinations are indicated by horizontal grey lines for depths approximating peak Photosynthetically Active Radiation (PAR; μmol photons m^-2^ s^-1^); and vertical grey lines for [O_2_] (μM). Data obtained from the Biological and Chemical Oceanography Data Management Office repository [43].

Ocean detections of proteins [43] mediating protein metabolism support this interpretation of distinct FtsH function across clades of *P*. *marinus* (Fig 7). Ribosome proteins annotated as from clade HLI (including MED4) were not detected at stations with low [O_2_], and near surface detections of ribosomes from clade HLI were far more frequent than detections at depth. Ribosome proteins from clade LLI (including NATL2A); clade LLII/III (including SS120) and clade LLIV (including MIT9313) show generally similar patterns vs. [O_2_] and depth, with at least some detections from stations with low [O_2_]. FtsH3, inferred to mediate PSI assembly, likewise shows a similar pattern between clade HLI and clade LLIV (Fig 7). Only clade HLI shows the presence of FtsH1 isoforms, inferred to partner with FtsH2, to mediate PSII repair, and then only in near-surface samples subject to higher light levels. Furthermore, even though clade IV grows (Fig 5B), and is detected in the ocean at low [O_2_] (Fig 2), no FtsH from clade IV, nor indeed FtsH from any clade, is detected at low [O_2_] (Fig 7), consistent with limited requirements for protein turnover under low [O_2_].

**Fig 8:**
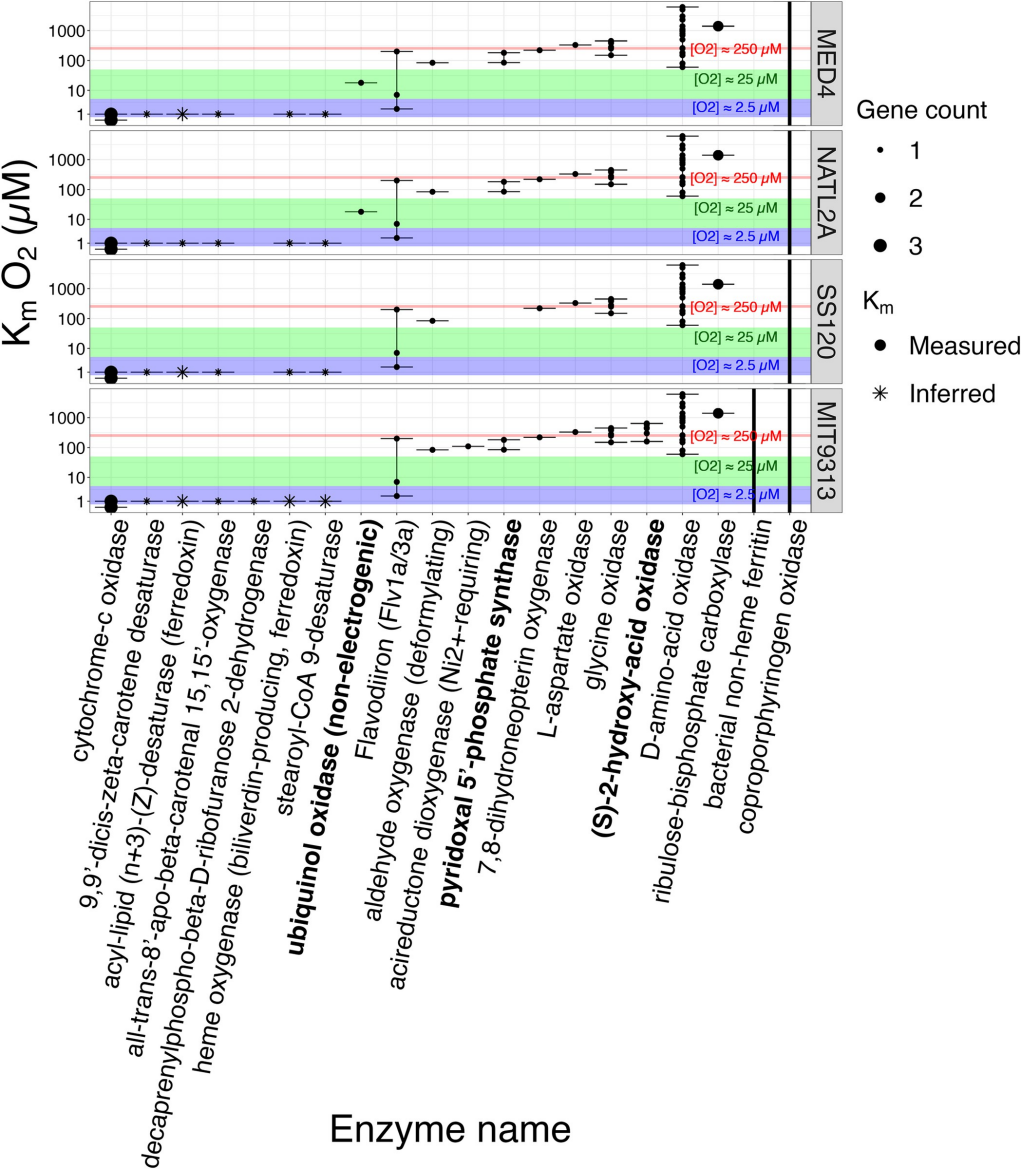
K_m_ values for oxygen metabolizing enzymes. The y-axis represents the log10 concentration of oxygen substrate (μM). The x-axis represents the oxygen metabolizing enzymes encoded in at least one of the *Prochlorococcus marinus* strains in this study. The *Prochlorococcus marinus* strains are indicated in rows. The solid circles represent K_m_ values from literature and the asterisks represent predicted values. Colours represent the gene counts. The red shaded area denotes a K_m_ oxygen concentration range from 230 to 280 μM. The green shaded area denotes a K_m_ oxygen concentration range from 5 to 50 μM. The blue shaded area denotes a K_m_ oxygen concentration range from 0.5 to 5 μM. The black bars show the minimum and maximum K_m_ values. Figure was generated using a filtered subset of the annotated phytoplankton gene sequences dataset from Omar *et al*. [62].

Fig 8 shows the measured or inferred K_m_ for [O_2_] for enzymes encoded by genes [62] from studied *P*. *marinus* strains, from clades HLI, LLI, LLII/III and LLIV. MED4 increases expression of alternative oxidase (‘ubiquinol oxidase (non electrogenic)’) to cope with changes in light [80], by dissipating electrons from the inter-system transport chain. The approximate K_m_ for [O_2_] of ~25 μM for ubiquinol oxidase (non electrogenic) (Fig 8) is comparable to the lower limit for growth of MED4 in our experiments (Fig 3B). We suggest that dependence upon ubiquinol oxidase excludes MED4 from low oxygen zones. The genome scan shows SS120 and MIT9313 lack the gene for ubiquinol oxidase (Fig 8), and therefore, lack this oxygen-dependent path to cope with changing excitation. Conversely, a gene encoding (S)-2-hydroxy-acid oxidase is encoded in the MIT9313 genome (Fig 8). (S)-2-hydroxy-acid oxidase catalyzes the reaction of 2-hydroxy acid with O_2_ to produce toxic H_2_O_2_ [81], with an approximate K_m_ for [O_2_] of ~250 μM. Growth at lower [O_2_] may thus protect MIT9313 from auto-intoxication from production of H_2_O_2_ by (S)-2-hydroxy-acid oxidase. We hypothesize that under 250 μM O_2_ and higher blue light, *P*. *marinus* MIT9313 suffered photoinhibition, resulting from the inactivation of PSII caused by the production of the reactive oxygen species, hydrogen peroxide. This photoinhibition is compounded by the limited inducible repair mechanism for PSII, due to the absence of inducible expression of FtsH 1 and 2 in *P*. *marinus* MIT9313 [48]. We hypothesize that under the conditions of our high light and 2.5 μM or 25 μM O_2_ experiments, the activity of the (S)-2-hydroxy-acid oxidase enzyme is suppressed. As a result, the catalyzed production of hydrogen peroxide is inhibited, leading to less PSII damage, allowing MIT9313 to avoid photoinhibition and circumvent its limitations on PSII repair, to exploit higher light. Fig 8 also shows that *P*. *marinus* SS120 is the only tested ecotype to lack the pyridoxal 5’-phosphate synthase enzyme. The pyridoxal 5’-phosphate synthase enzyme is an important cofactor in the biosynthesis of vitamin B_6_ [82]. Vitamin B_6_ is a potential antioxidant and can effectively quench singlet oxygen [83]. The absence of the pyridoxal 5’-phosphate synthase enzyme may explain why *P*. *marinus* SS120 does not grow as well as *P*. *marinus* MIT9313, when exposed to high light stress even under 25 μM O_2_, and not at all under 2.5 μM O_2_ (Fig 4).

**Fig 9:**
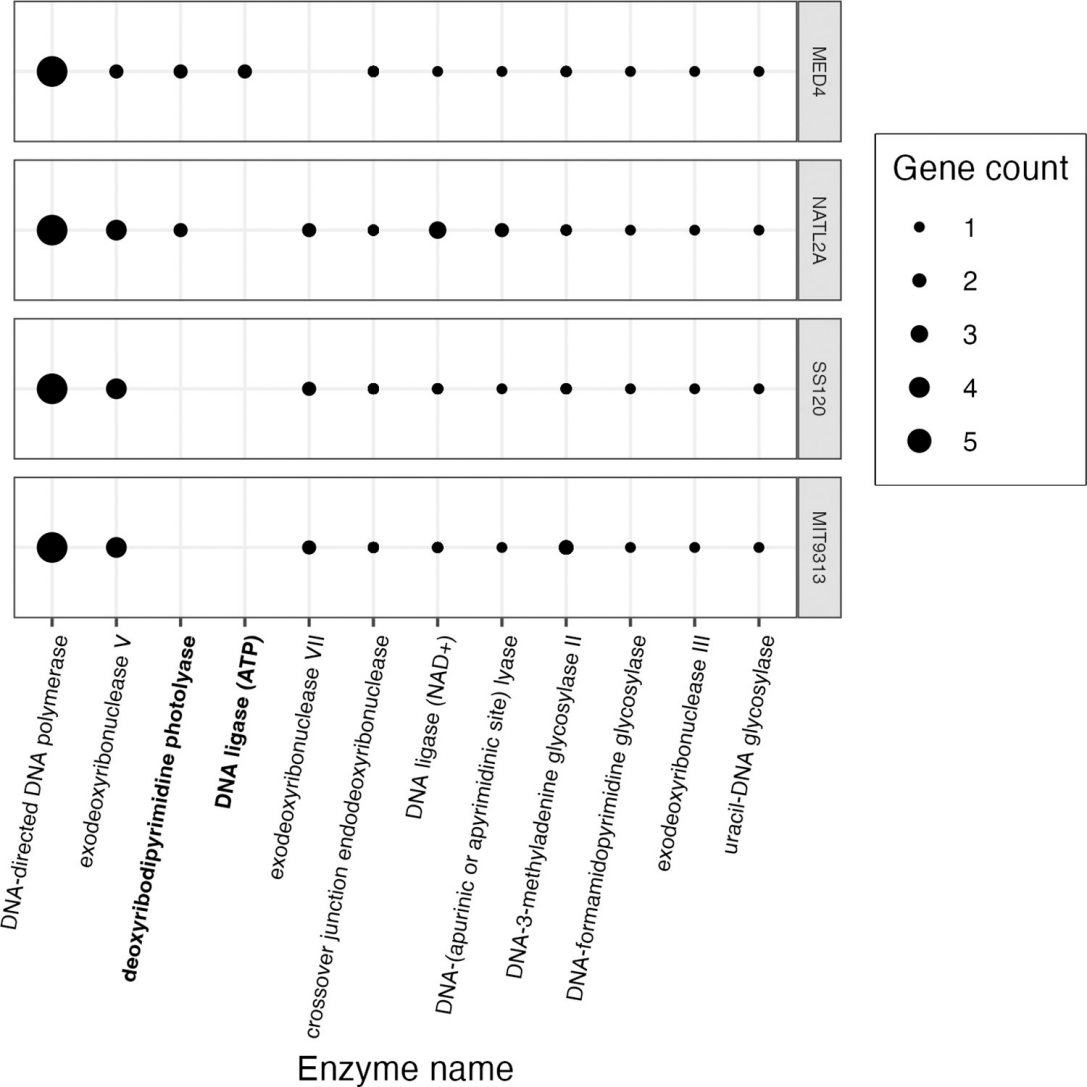
Genes encoding DNA repair enzymes. The y-axis represents *Prochlorococcus marinus* strains. The x-axis represents enzymes encoded for DNA repair found in at least one *Prochlorococcus marinus* strain in this study. Point size indicate gene counts. Figure was generated using a filtered subset of the annotated phytoplankton gene sequences dataset from Omar *et al*. [62].

To gain insights into the distinct growth rate responses across strains, particularly the responses to different [O_2_], we used a dataset of annotated phytoplankton genomes from Omar *et al*. [62]. We tabulated those genes encoding enzymes involved in DNA repair, present in at least one of the *P*. *marinus* study strains (Fig 9).

*Prochlorococcus marinus* NATL2A and MED4 possess the largest, most complete suites of genes encoding DNA repair enzymes. *Prochlorococcus marinus* MED4 and NATL2A both possess a gene encoding deoxyribodipyrimidine photolyase (Figs 9 and S5), which, when activated by blue light, repairs DNA damaged by UV light [84]. *Prochlorococcus marinus* MED4 was also the only strain to possess a gene encoding DNA repair ligase, which uses ATP as a cofactor for DNA repair.

Conversely, *P*. *marinus* SS120 and MIT9313 lack genes encoding deoxyribodipyrimidine photolyase and DNA repair ligase (ATP), which may explain, in part, why these two strains cannot tolerate growth under full 250 μM [O_2_] and high light, as found at the ocean surface. *Prochlorococcus marinus* SS120 and MIT9313 do, in contrast, tolerate light levels representative of the near surface ocean at 25 μM [O_2_]. Our growth rate experiments did not include wavebands in the UV range, so we do not know whether the absence of deoxyribodipyrimidine photolyase would inhibit the growth rates of *P*. *marinus* SS120 and MIT9313 under low [O_2_] if exposed to full spectrum, ocean surface light. The protective effects of lower [O_2_], allowing these strains to grow at higher light, likely relates in part to suppression of DNA damage when generation of Reactive Oxygen Species (ROS) is suppressed at lower [O_2_]. NATL2A, a clade LLI, has been found near the ocean surface during deep ocean mixing [85] events. Malmstrom *et al*. [85] indeed attribute NATL2A tolerance of short exposures of high light to presence of genes encoding photolyase, also found in HL clades. The presence of deoxyribodipyrimidine photolyase but absence of DNA repair ligase (ATP) supports why NATL2A tolerates limited exposure to high light, but is unable to fully repair damaged DNA. *Prochlorococcus marinus* are highly susceptible to hydrogen peroxide (H_2_O_2_) toxicity as they lack genes which scavenge H_2_O_2_ molecules [51]. The small cell size of *P*. *marinus* allow the ROS, H_2_O_2_, to cross the cell membrane [86]; however, accumulation of extracellular H_2_O_2_ remains toxic to *P*. *marinus* [51,52].

Diverse *P*. *marinus* strains [7] differentially exploit potential photoregimes, both at the surface and deeper in the water column. Some *P*. *marinus* strains grow under low oxygen environments, similar to OMZ. The LLII and LLIV clade representatives we tested can function as ‘HL’ in oxygen environments of 25 μM, and as low as 2.5 μM, in the case of MIT9313.

West *et al*. [21] and Malmstrom *et al*. [85] found that decreased abundances of the LL clades corresponded to increased depth of the surface mixed layer. Malmstrom *et al*. [85] attributes the transport of LL ecotypes to the surface and consequent exposure to photoinhibitory high light levels as the reason for low cell abundances with increased mixed layer depth. West *et al*. [21] found the depth of the mixed layer strongly influenced the depth transition from HL to LL clades, but that factors other than light levels may influence the variations in the upper and lower depth limits of these ecotypes. We hypothesize that low cell abundances of LL ecotypes in the mixed layer is likely driven in part by increased [O_2_], and it is [O_2_] that constrains LL clades to deeper waters, not necessarily the light level. We found that under 25 μM O_2_ representatives of ‘LL’ clades, SS120 and MIT9313, actually tolerate approximately 1.0 x 10^6^ μmol photons m^-2^ d^-1^ of PUR (S4E and S4H Fig), comparable to the representative HL clade, MED4 which also exhibited growth rate saturation at the same cumulative diel PUR of 1.0 x 10^6^ μmol photons m^-2^ d^-1^ (S4A and S4B Fig). Growth under lower O_2_ allowed MIT9313 to substantially increase its exploitation of higher diel PUR (S4I Fig).

**Fig 10:**
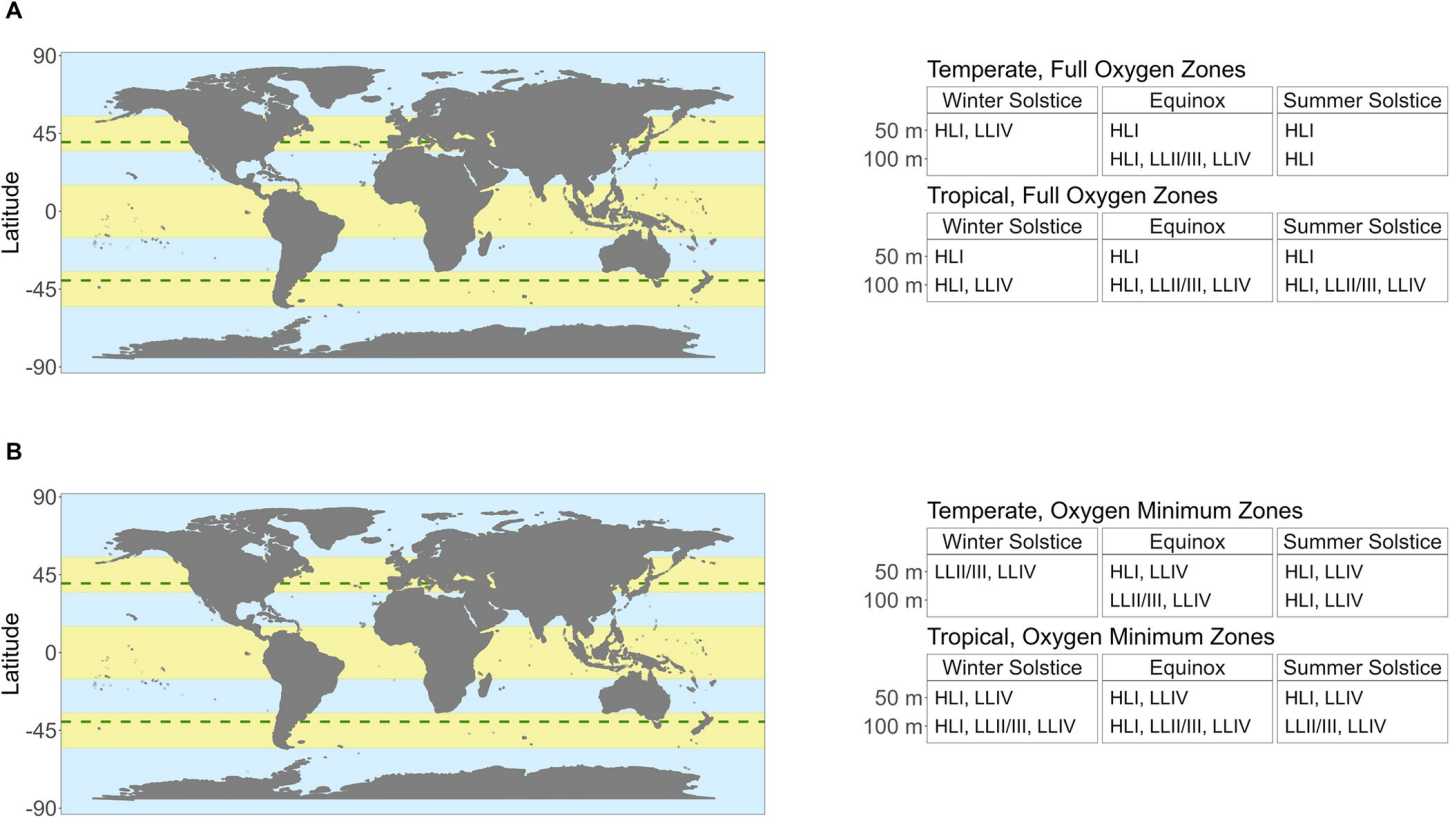
Potential Future Niches for *Prochlorococcus* clades in a warming ocean. The maps shows current latitudinal distribution limits for *Prochlorococcus* (between green dashed lines), along with tropical and temperate latitudinal regions (yellow bands). Light levels, photoperiods and spectral bands, potentially permissive for growth of *Prochlorococcus* clades are determined by the interactions of latitude, season and depth attenuation of light. **A.** Potential clade occupancies under hypothetical Full Oxygen Zones (250 μM O_2_, not shown on map) are shown for 10–50 m and 100 m depths, in temperate vs. tropical regions, under depth-resolved photoperiods, approximating Winter Solstice, Equinox and Summer Solstice. Clade HLI is excluded from temperate depths by short photoperiods at Winter Solstice. Clades LLII/III and LLIV are excluded from shallow depths by excessive light. **B.** Oxygen Minimum Zones (25 μM O_2_, not shown on map) would drastically alter these potential occupancy patterns by excluding clade HLI from the temperate winter solstice at all depths, and by expanding the exploitation of higher light zones by Clade LLIV. Potential clade occupancies for the southern temperate zone mirror the northern temperate zone occupancies.

Potential niche expansions of clades of *P*. *marinus* into temperate regions will vary depending on the season, and the depth, which interact to govern the growth limiting factors of peak PAR levels and underwater photoperiods (Fig 10). Temperate summer delivers ~12–14 hours of light, depending upon depth, above a photic threshold of 20 μmol photons m^-2^ s^-1^, while temperate spring/fall delivers ~9–11 hours; photoperiod ranges permissive for growth of all three *P*. *marinus*. In contrast temperate winter delivers shorter photoperiods which may exclude clade HLI in deeper regions. In parallel, excess peak PAR will exclude clades LLII/III and LLIV from most shallow niches, even under permissive temperatures.

Expansion of OMZ would drastically alter these patterns of potential niche extensions, by further restricting occurrences of clade HLI, but allowing clade LLIV to exploit a much wider range of lights, extending towards near-surface niches in both tropical and temperate zones, under permissive temperatures.

## Summary and conclusions

We analyzed growth rates for *P*. *marinus* clade HLI usually found near the ocean surface; clade LLII/III found deeper in the water column; and clade LLIV also found in deep oceans, including current OMZ, under a matrix of spectral wavebands, irradiances, photoperiods and oxygen concentrations approximating present day and hypothetical future niches.

*Prochlorococcus marinus* MED4 requires more than 4 h of light per day; thus this strain will not exploit habitats typical of temperate winters at light attenuated depths, even if water temperatures warm into the clade HLI tolerance range. MED4 is also excluded from the lowest oxygen habitats of 2.5 μM O_2_, but can, grow under OMZ regions with 25 μM O_2_. Genomic (Fig 8) and transcriptional analyses [80] suggest MED4 is excluded from growth below ~25 μM O_2_ because it relies upon a ubiquinol oxidase, non-electrogenic, to maintain oxidation/reduction balance in the intersystem electron transport chain, with a K_m_ for [O_2_] of ~25 μM O_2_. On the other hand, MED4 shows inducible expression of FtsH isoforms [48], to counter photoinactivation of PSII under higher PAR and [O_2_] environments. However, photoinactivation imposes an increased cost of growth upon MED4, since growth under red light, to lower photoinactivation of PSII [60], allows MED4 to achieve faster growth per absorbed photon than growth under blue light. TARA Oceans Project data [22] indeed reported presence of *P*. *marinus* MED4-like genomes at depths ranging from 5 m to 90 m, representing high to low blue light levels, in the Pacific South East Ocean. Our growth findings are consistent with Fig 2 showing PSII proteins annotated as MED4, clade HLI, at depths up to 200 meters, with O_2_ of ~15 μM.

*Prochlorococcus marinus* SS120, a clade LLII/III representative, showed an interactive inhibition of growth by oxygen and cumulative diel PUR, with a higher tolerance for higher cumulative diel PUR under 25 μM O_2_, compared to 250 μM O_2_ (S4 Fig). Thus, SS120 can exploit higher PAR environments, within OMZ. SS120 is likely excluded from the combination of higher [O_2_] and higher PAR by genomic limitations on capacity for DNA repair (Fig 9), and possibly by limited capacity for synthesis of reactive oxygen quenchers (Fig 8). Our growth results are supported by Lavin *et al*. [12] who found evidence of clades LLII/III and LLIV, using terminal restriction fragment length polymorphism analyses, at depths above 40 m, where light levels are higher, within OMZ, and by Fig 2 showing PSII protein subunits annotated as derived from SS120 at all depths ranging from 20 to 200 m and all [O_2_] in an OMZ of the tropical North Pacific Ocean. SS120 grew under photoperiods longer than 4 h and showed increasing growth rate with increasing photoperiods, and so has the potential to thrive in deep temperate zones, specifically during the spring, summer, and fall seasons when the duration of daylight exceeds 4 h, if [O_2_] are near surface saturation of about 250 μM. Under lower oxygen levels of 25 μM, SS120 can also potentially exploit a 4 h photoperiod in the blue waveband, and thus has the potential to inhabit a potential warmed, deep, temperate OMZ, during the winter season.

*Prochlorococcus marinus* MIT9313, a clade LLIV representative, shows potential to inhabit future warmer temperate zones year-round, as it grows even under a 4 h photoperiod, expected in winter, or at light-attenuated depths. MIT9313 demonstrates an unexpected tolerance to higher light levels and cumulative diel PUR, but only under low oxygen conditions of 25 μM and 2.5 μM (Fig 5A), enabling MIT9313 to grow in OMZ, even at depths closer to the surface. MIT9313 carries a gene encoding (S)-2-hydroxy-acid oxidase [81], with a K_m_ for [O_2_] of ~250 μM (Fig 8), which produces H_2_O_2_. Growth at lower [O_2_] may protect MIT9313 from auto-intoxication from production of H_2_O_2_. We hypothesize that under 250 μM O_2_ and higher blue light, *P*. *marinus* MIT9313 suffers photoinhibition, resulting in part from the inactivation of PSII caused by the production of H_2_O_2_. This photoinhibition is compounded by limited inducible repair for PSII, due to the absence of FtsH 1 and 2 expression in *P*. *marinus* MIT9313 [48]. MIT9313 shows remarkable ability to thrive under very low [O_2_], potentially allowing it to expand into broader ecological niches. Our results are supported by Fig 2 showing protein subunits derived from MIT9313 detected frequently at depths > 120 m in regions where O_2_ was 15 μM. Bagby and Chisholm [87] suggest that O_2_ has a protective role in *P*. *marinus* under lower carbon dioxide environments when carbon fixation is limited. The deep water environments typical for MIT9313 are relatively nutrient rich, and *P*. *marinus* take up and metabolize various sugars [88–90] and amino acids [91]. In future work could test whether MIT9313 is using photosynthesis to drive CO_2_ fixation in low O_2_ environments, or whether PSII generation of O_2_ acts as an electron sink for respiration, using ATP for maintenance and to take up nutrients from the surroundings. Partensky *et al*. [17] indeed found that in the low-light conditions found in the OMZ, MED4, SS120 and MIT9313 all became net O_2_ consumers, suggesting that low light levels cause the respiratory chain to consume more O_2_ than the photosynthetic electron transport chain generates, thus contributing to maintenance of the low O_2_ environment.

In warming oceans, *P*. *marinus* clades will differentially expand into new regions. Competition among clades will be driven not simply by light levels, but by their differing capacities to tolerate and exploit combinations of photoperiods, light levels, and [O_2_]. Clade HLI (including MED4) is excluded from short photoperiod regimes, typical of temperate winters at light attenuated depths. In contrast, clade LLIV (including MIT9313) may exploit higher light niches under expanding OMZ conditions, where low O_2_ relieves the stresses of oxidative stress and PSII photoinhibition.

## Supporting information

S1 Fig PSI MCMIX-OD Multicultivator. Spectral waveband, light level and photoperiod are individually controlled for each culture tube. Real time Optical Density (OD) measurements eliminate intrusive subsampling of cultures. The temperature of culture tubes are collectively controlled via heating or cooling of the aquarium water. Gas with specific oxygen concentrations is bubbled through a humidifier and passed through a 0.2 μm filter.
(TIF)

S2 Fig Fitting chlorophyll specific growth rate for each tube in the Multicultivator. The x-axis is time in hours (h). The left y-axis is chlorophyll proxy optical density (OD_680_—OD_720_; *Δ*OD) The right y-axis is the Photosynthetically Active Radiation (PAR; μmol photons m^-2^ s^-1^) levels; colours represent the imposed spectral waveband: 450 nm (blue points) or 660 nm (red points). The green points are *Δ*OD measurements taken every 5 minutes. The black lines are logistic growth rate curves fit using a nonlinear model regression (R package, minpack.lm). The gold points are the residuals of the fit. Meta data associated with each Multicultivator tube are in columns.
(TIF)

S3 Fig **Normalized absorbance, emission and Photosynthetically Usable Radiation spectra for *Prochlorococcus marinus* MED4 (A-C); SS120 (D-F); MIT9313 (G-I) grown under three emission wavebands. (A,D,G)** Growth light emission spectra from the White LED (normalized to 439 nm; dotted black line); whole cell absorbance spectra (normalized to absorbance maxima between 400 nm and 460 nm; dashed purple line); and calculated PUR spectra (solid black line and shaded grey). **(B,E,H)** Growth light emission spectra at 660 nm (normalized to 647 nm; dotted red line); whole cell absorbance spectra (normalized to absorbance maxima between 400 nm and 460 nm; dashed purple line); and calculated PUR spectra (solid black line and shaded red). **(C,F,I)** Growth light emission spectra at 450 nm (normalized to 441 nm; dotted blue line); whole cell absorbance spectra (normalized to absorbance maxima between 400 nm and 460 nm; dashed purple line); and calculated PUR spectra (solid black line and shaded blue). Photosynthetically Active Radiation (PAR; μmol photons m^-2^ s^-1^) and calculated Photosynthetically Usable Radiation (PUR; μmol photons m^-2^ s^-1^) levels are indicated.
(TIF)

S4 Fig Chlorophyll specific growth rate (d^-1^) vs. cumulative diel Photosynthetically Usable Radiation (PUR, μmol photons m^-2^ d^-1^). Rows separate data from levels of imposed dissolved O_2_ concentrations as 250 μM, 25 μM and 2.5 μM. Columns separate data from strains; MED4 (A-C), SS120 (D-F) and MIT9313 (G-I). Shapes show the imposed photoperiod (h); 4 h (solid square), 8 h (solid diamond), 12 h (solid circle), 16 h (solid upright triangle). Symbol colours show the spectral waveband for growth; white LED (black symbols), 660 nm (red symbols), and 450 nm (blue symbols). Large symbols show mean of growth rate from logistic curve fits; small symbols show values for replicate determinations, if any. Harrison and Platt [54] 4 parameter model fit to data pooled for each combination of strain and dissolved oxygen shown with solid lines. Separate models fit to photoperiod data and shown if significantly different (*P* value < 0.05) from the pooled model using one-way ANOVA; 4 h (long dashed line); 8 h (dotted line); 12 h (dashed line); and 16 h (dot dashed line).
(TIF)

S5 Fig *Prochlorococcus marinus* genes encoding enzymes activated or inhibited by light. The y-axis represents *Prochlorococcus marinus* strains. The x-axis represents enzymes encoding light-dependent enzymes found in at least one *Prochlorococcus marinus* strain in this study. Point size indicate gene counts. Figure was generated using a filtered subset of the annotated phytoplankton gene sequences dataset from Omar *et al*. [62].
(TIF)

S1 Table Enzymes shown in Figs 8, 9 and S5 with their Enzyme Commission numbers (EC) and Kegg Orthology (KO). (DOCX)

S2 Table The maximum growth rate, μ_max_ (d^-1^) achieved for each strain under each [O_2_], with the corresponding photoperiod, PAR level and spectral waveband. (DOCX)
