# Supplementary figures and images for: Prochlorococcus marinus responses to light and oxygen
Source: PLoS One. 2024 Jul 22;19(7):e0307549. doi: 10.1371/journal.pone.0307549 (PMC11262661; doi:10.1371/journal.pone.0307549)

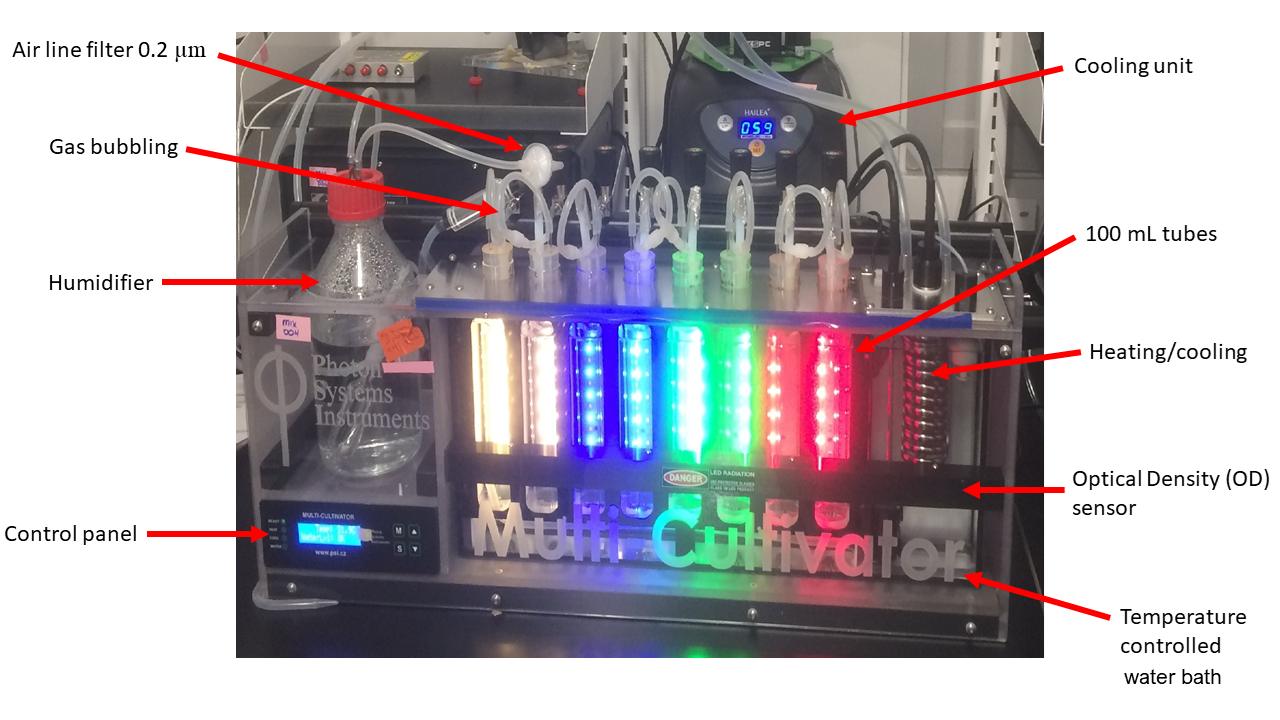

Supplement: S1 Fig — Spectral waveband, light level and photoperiod are individually controlled for each culture tube. Real time Optical Density (OD) measurements eliminate intrusive subsampling of cultures. The temperature of culture tubes are collectively controlled via heating or cooling of the aquarium water. Gas with specific oxygen concentrations is bubbled through a humidifier and passed through a 0.2 μm filter. (TIF) [file pone.0307549.s001.tif]

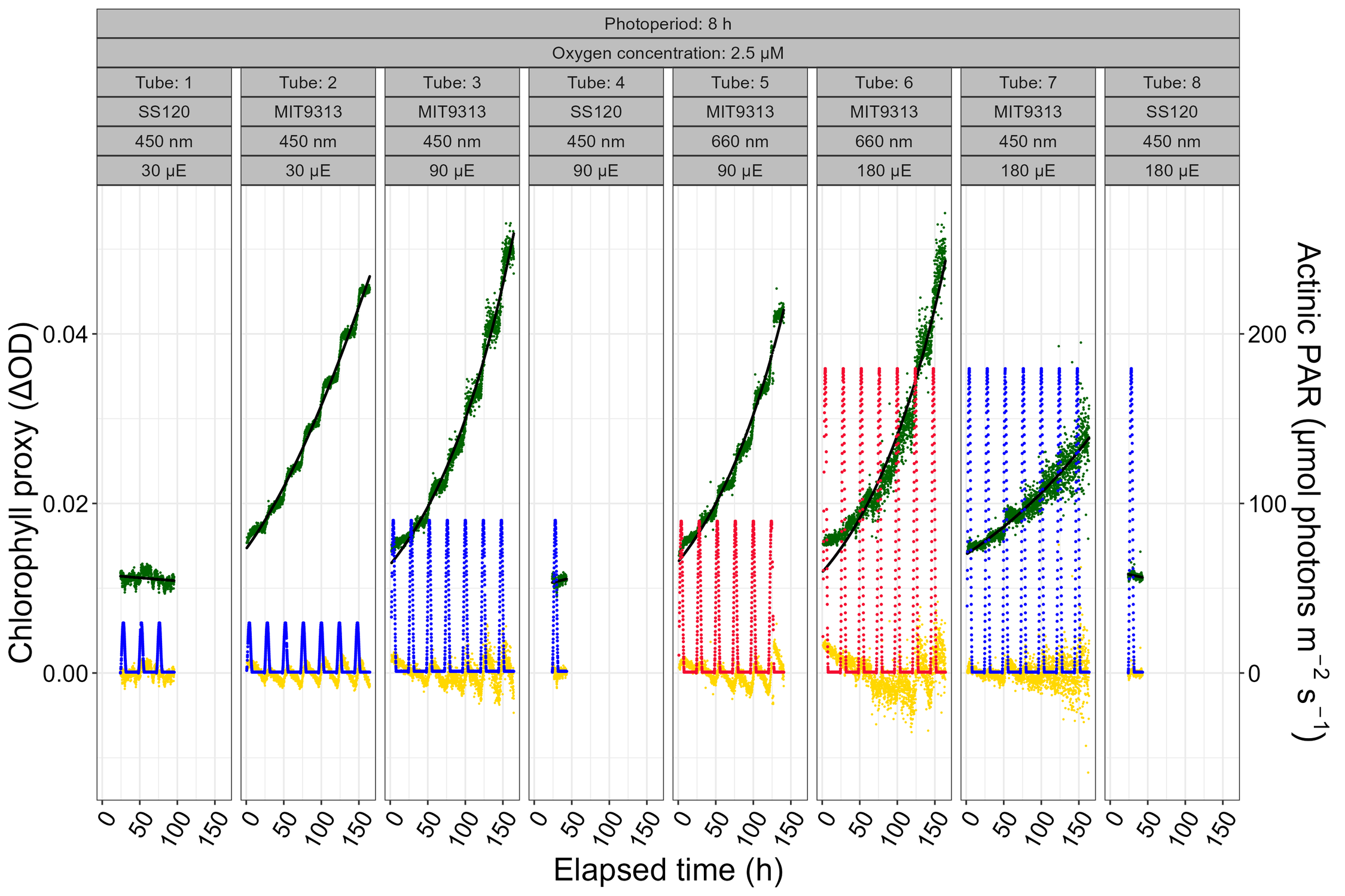

Supplement: S2 Fig — The x-axis is time in hours (h). The left y-axis is chlorophyll proxy optical density (OD680—OD720; ΔOD) The right y-axis is the Photosynthetically Active Radiation (PAR; μmol photons m-2 s-1) levels; colours represent the imposed spectral waveband: 450 nm (blue points) or 660 nm (red points). The green points are ΔOD measurements taken every 5 minutes. The black lines are logistic growth rate curves fit using a nonlinear model regression (R package, minpack.lm). The gold points are the residuals of the fit. Meta data associated with each Multicultivator tube are in columns. (TIF) [file pone.0307549.s002.tif]

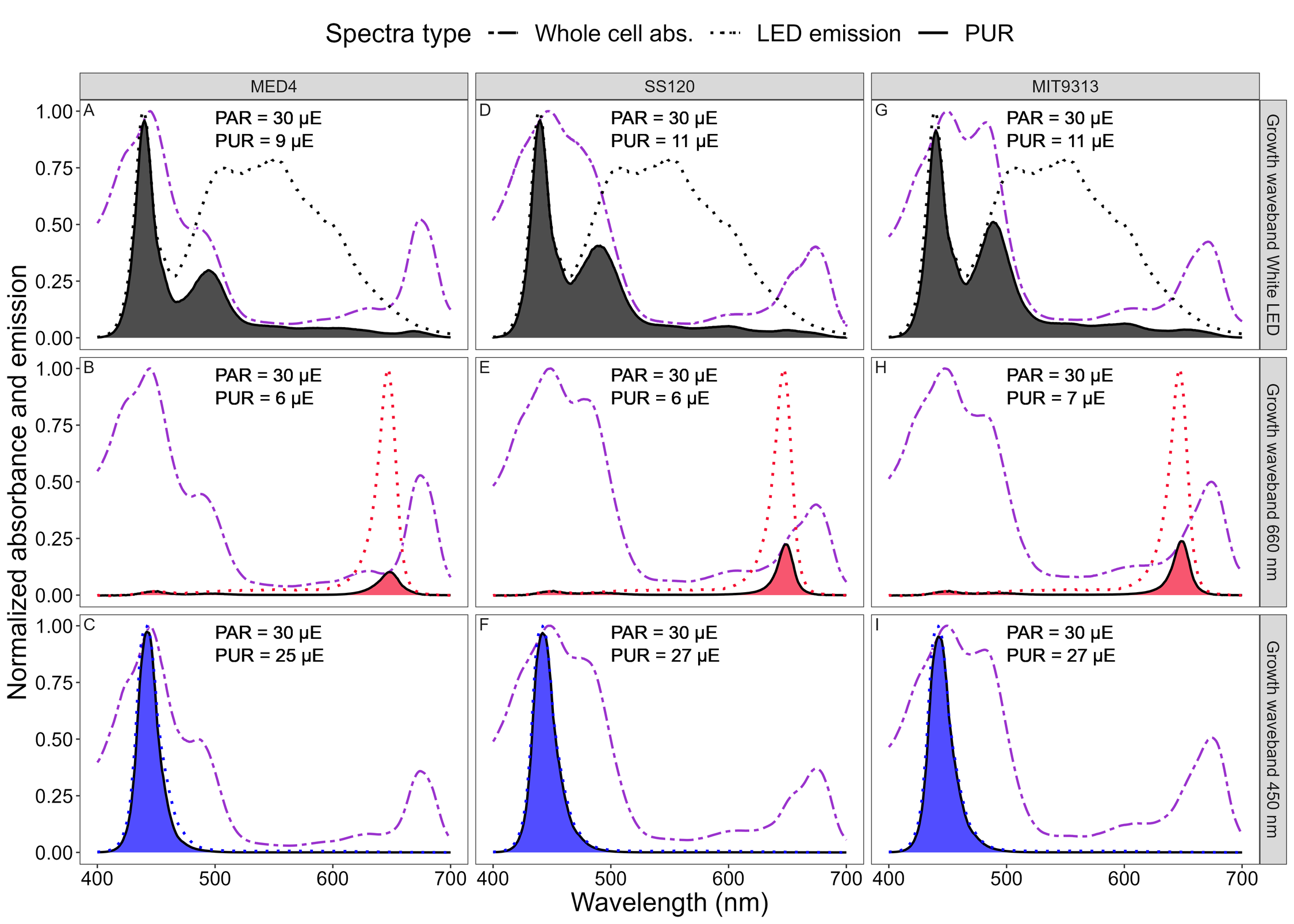

Supplement: S3 Fig — Normalized absorbance, emission and Photosynthetically Usable Radiation spectra for Prochlorococcus marinus MED4 (A-C); SS120 (D-F); MIT9313 (G-I) grown under three emission wavebands. (A,D,G) Growth light emission spectra from the White LED (normalized to 439 nm; dotted black line); whole cell absorbance spectra (normalized to absorbance maxima between 400 nm and 460 nm; dashed purple line); and calculated PUR spectra (solid black line and shaded grey). (B,E,H) Growth light emission spectra at 660 nm (normalized to 647 nm; dotted red line); whole cell absorbance spectra (normalized to absorbance maxima between 400 nm and 460 nm; dashed purple line); and calculated PUR spectra (solid black line and shaded red). (C,F,I) Growth light emission spectra at 450 nm (normalized to 441 nm; dotted blue line); whole cell absorbance spectra (normalized to absorbance maxima between 400 nm and 460 nm; dashed purple line); and calculated PUR spectra (solid black line and shaded blue). Photosynthetically Active Radiation (PAR; μmol photons m-2 s-1) and calculated Photosynthetically Usable Radiation (PUR; μmol photons m-2 s-1) levels are indicated. (TIF) [file pone.0307549.s003.tif]

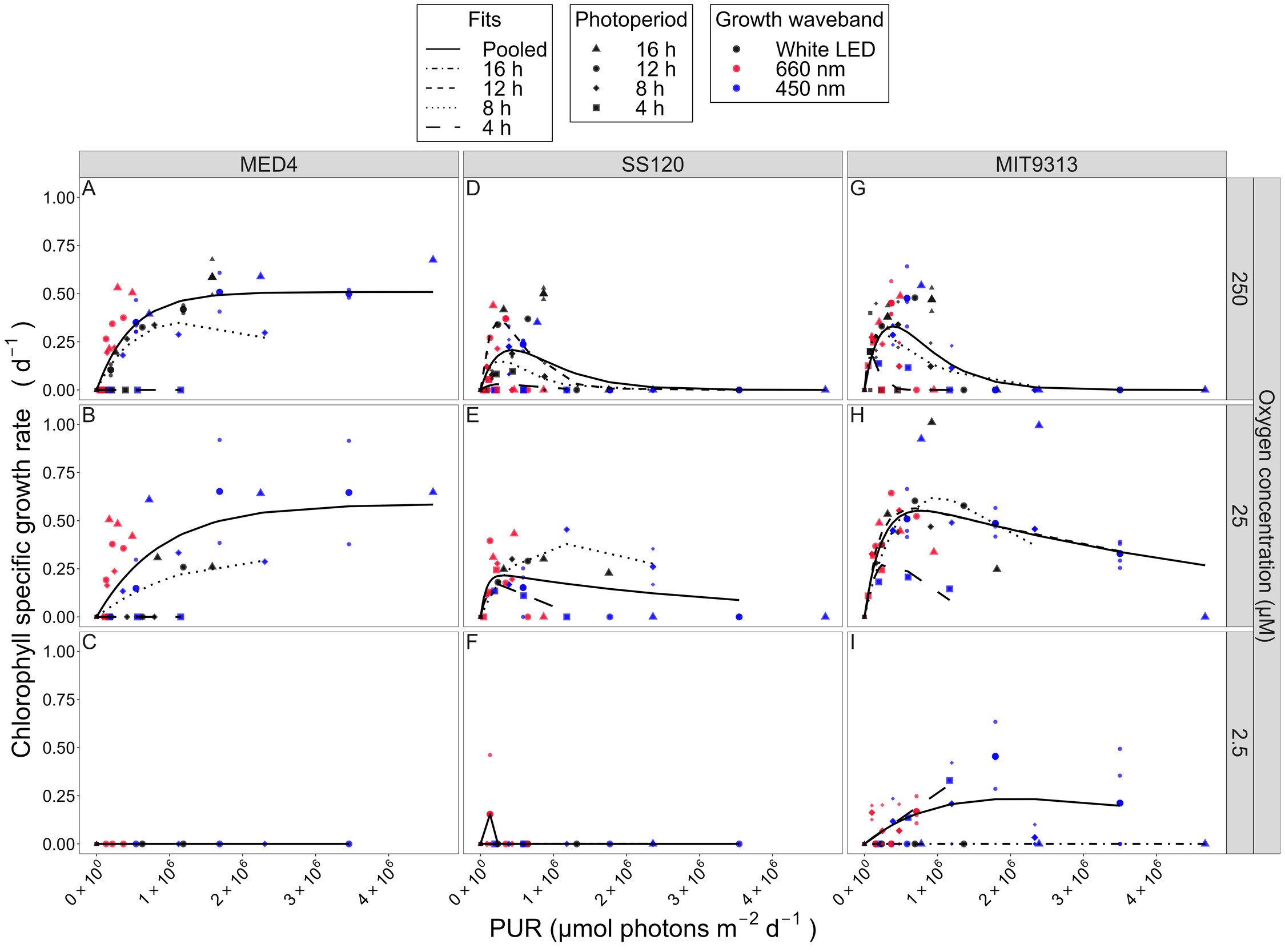

Supplement: S4 Fig — Rows separate data from levels of imposed dissolved O2 concentrations as 250 μM, 25 μM and 2.5 μM. Columns separate data from strains; MED4 (A-C), SS120 (D-F) and MIT9313 (G-I). Shapes show the imposed photoperiod (h); 4 h (solid square), 8 h (solid diamond), 12 h (solid circle), 16 h (solid upright triangle). Symbol colours show the spectral waveband for growth; white LED (black symbols), 660 nm (red symbols), and 450 nm (blue symbols). Large symbols show mean of growth rate from logistic curve fits; small symbols show values for replicate determinations, if any. Harrison and Platt [54] 4 parameter model fit to data pooled for each combination of strain and dissolved oxygen shown with solid lines. Separate models fit to photoperiod data and shown if significantly different (P value < 0.05) from the pooled model using one-way ANOVA; 4 h (long dashed line); 8 h (dotted line); 12 h (dashed line); and 16 h (dot dashed line). (TIF) [file pone.0307549.s004.tif]

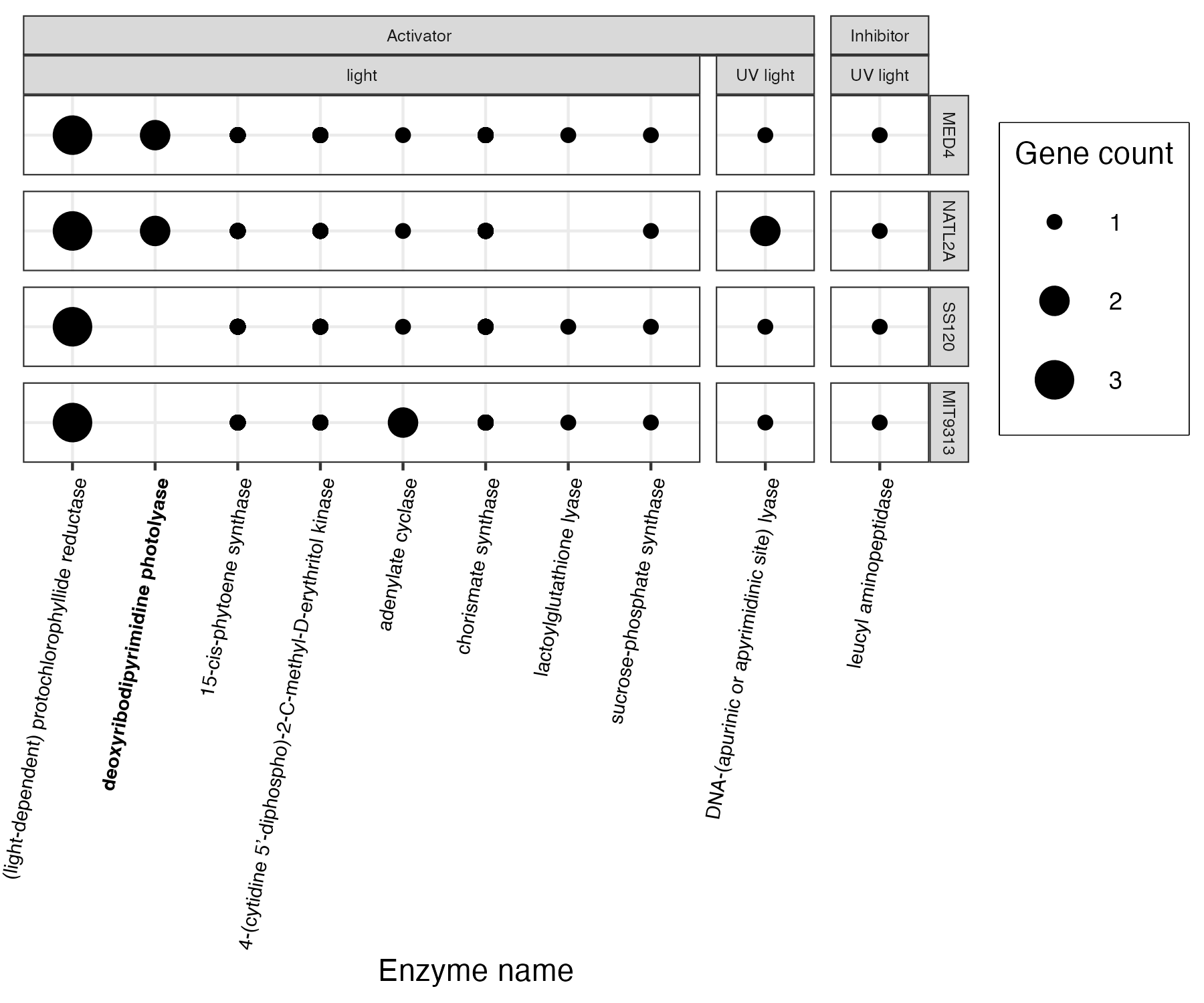

Supplement: S5 Fig — The y-axis represents Prochlorococcus marinus strains. The x-axis represents enzymes encoding light-dependent enzymes found in at least one Prochlorococcus marinus strain in this study. Point size indicate gene counts. Figure was generated using a filtered subset of the annotated phytoplankton gene sequences dataset from Omar et al. [62]. (TIF) [file pone.0307549.s005.tif]
