# Supplementary material for: Prochlorococcus marinus responses to light and oxygen
Source: PLoS One. 2024 Jul 22;19(7):e0307549. doi: 10.1371/journal.pone.0307549 (PMC11262661; doi:10.1371/journal.pone.0307549)
Supplement: S1 Table — (DOCX) [file pone.0307549.s006.docx]

| Enzyme Name | EC | Kegg Orthology |
| --- | --- | --- |
| quinate dehydrogenase | 1.1.1.24 | K09484 |
| pyranose oxidase | 1.1.3.10 | K23272 |
| L-sorbose oxidase | 1.1.3.11 | NA |
| pyridoxine 4-oxidase | 1.1.3.12 | K18607 |
| alcohol oxidase | 1.1.3.13 | K17066 |
| (S)-2-hydroxy-acid oxidase | 1.1.3.15 | K00104 |
| (S)-2-hydroxy-acid oxidase | 1.1.3.15 | K11517 |
| ecdysone oxidase | 1.1.3.16 | K10724 |
| choline oxidase | 1.1.3.17 | K17755 |
| secondary-alcohol oxidase | 1.1.3.18 | NA |
| 4-hydroxymandelate oxidase (decarboxylating) | 1.1.3.19 | NA |
| long-chain-alcohol oxidase | 1.1.3.20 | K17756 |
| long-chain-alcohol oxidase | 1.1.3.20 | NA |
| glycerol-3-phosphate oxidase | 1.1.3.21 | K00105 |
| thiamine oxidase | 1.1.3.23 | NA |
| hydroxyphytanate oxidase | 1.1.3.27 | NA |
| nucleoside oxidase | 1.1.3.28 | NA |
| polyvinyl-alcohol oxidase | 1.1.3.30 | NA |
| D-arabinono-1,4-lactone oxidase | 1.1.3.37 | K00107 |
| vanillyl-alcohol oxidase | 1.1.3.38 | K20153 |
| nucleoside oxidase (H2O2-forming) | 1.1.3.39 | NA |
| glucose oxidase | 1.1.3.4 | NA |
| D-mannitol oxidase | 1.1.3.40 | NA |
| alditol oxidase | 1.1.3.41 | K00594 |
| prosolanapyrone-II oxidase | 1.1.3.42 | K20550 |
| aclacinomycin-N oxidase | 1.1.3.45 | K15949 |
| 4-hydroxymandelate oxidase | 1.1.3.46 | K16422 |
| 5-(hydroxymethyl)furfural oxidase | 1.1.3.47 | K16873 |
| 3-deoxy-alpha-D-manno-octulosonate 8-oxidase | 1.1.3.48 | K19714 |
| hexose oxidase | 1.1.3.5 | K21840 |
| cholesterol oxidase | 1.1.3.6 | K03333 |
| aryl-alcohol oxidase | 1.1.3.7 | NA |
| L-gulonolactone oxidase | 1.1.3.8 | K00103 |
| galactose oxidase | 1.1.3.9 | K04618 |
| glycerol oxidase | 1.1.3.B4 | NA |
| (S)-2-hydroxyglutarate dehydrogenase | 1.1.5.13 | NA |
| decaprenylphospho-beta-D-ribofuranose 2-dehydrogenase | 1.1.98.3 | NA |
| cellobiose dehydrogenase (acceptor) | 1.1.99.18 | NA |
| glucooligosaccharide oxidase | 1.1.99.B3 | NA |
| catechol oxidase | 1.10.3.1 | K00422 |
| ubiquinol oxidase (non-electrogenic) | 1.10.3.11 | K17893 |
| grixazone synthase | 1.10.3.15 | K20204 |
| superoxide oxidase | 1.10.3.17 | K12262 |
| laccase | 1.10.3.2 | K00421 |
| laccase | 1.10.3.2 | K05909 |
| L-ascorbate oxidase | 1.10.3.3 | K00423 |
| L-ascorbate oxidase | 1.10.3.3 | NA |
| o-aminophenol oxidase | 1.10.3.4 | K20204 |
| o-aminophenol oxidase | 1.10.3.4 | K20219 |
| 3-hydroxyanthranilate oxidase | 1.10.3.5 | NA |
| rifamycin-B oxidase | 1.10.3.6 | NA |
| catechol 1,2-dioxygenase | 1.13.11.1 | K03381 |
| 7,8-dihydroxykynurenate 8,8a-dioxygenase | 1.13.11.10 | NA |
| tryptophan 2,3-dioxygenase | 1.13.11.11 | K00453 |
| linoleate 13S-lipoxygenase | 1.13.11.12 | K00454 |
| 2,3-dihydroxybenzoate 3,4-dioxygenase | 1.13.11.14 | K10621 |
| 3,4-dihydroxyphenylacetate 2,3-dioxygenase | 1.13.11.15 | K00455 |
| 3-carboxyethylcatechol 2,3-dioxygenase | 1.13.11.16 | K05713 |
| indole 2,3-dioxygenase | 1.13.11.17 | NA |
| persulfide dioxygenase; | 1.13.11.18 | K17725 |
| cysteamine dioxygenase | 1.13.11.19 | K10712 |
| catechol 2,3-dioxygenase | 1.13.11.2 | K00446 |
| catechol 2,3-dioxygenase | 1.13.11.2 | K07104 |
| 4-hydroxyphenylpyruvate dioxygenase | 1.13.11.27 | K00457 |
| protocatechuate 3,4-dioxygenase | 1.13.11.3 | K00448 |
| protocatechuate 3,4-dioxygenase | 1.13.11.3 | K00449 |
| arachidonate 15-lipoxygenase | 1.13.11.33 | K00460 |
| arachidonate 15-lipoxygenase | 1.13.11.33 | K08022 |
| arachidonate 15-lipoxygenase | 1.13.11.33 | K19246 |
| arachidonate 5-lipoxygenase | 1.13.11.34 | K00461 |
| acireductone dioxygenase (Ni2+-requiring) | 1.13.11.53 | K08967 |
| linolenate 9R-lipoxygenase | 1.13.11.61 | K18031 |
| all-trans-8’-apo-beta-carotenal 15,15’-oxygenase | 1.13.11.75 | K00464 |
| 7,8-dihydroneopterin oxygenase | 1.13.11.81 | K01633 |
| peptide-aspartate beta-dioxygenase | 1.14.11.16 | K00476 |
| taurine dioxygenase | 1.14.11.17 | K03119 |
| procollagen-proline 4-dioxygenase | 1.14.11.2 | K00472 |
| nitric oxide dioxygenase | 1.14.12.17 | K05916 |
| salicylate 1-monooxygenase | 1.14.13.1 | K00480 |
| cyclohexanone monooxygenase | 1.14.13.22 | K03379 |
| violacein synthase | 1.14.13.224 | K20090 |
| L-lysine N6-monooxygenase (NADPH) | 1.14.13.59 | K03897 |
| magnesium-protoporphyrin IX monomethyl ester (oxidative) cyclase | 1.14.13.81 | K04035 |
| kynurenine 3-monooxygenase | 1.14.13.9 | K00486 |
| unspecific monooxygenase | 1.14.14.1 | K00490 |
| dimethylsulfone monooxygenase | 1.14.14.35 | K17228 |
| heme oxygenase (biliverdin-producing, ferredoxin) | 1.14.15.20 | K21480 |
| choline monooxygenase | 1.14.15.7 | K00499 |
| stearoyl-CoA 9-desaturase | 1.14.19.1 | K00507 |
| acyl-lipid (n+3)-(Z)-desaturase (ferredoxin) | 1.14.19.23 | K10255 |
| tetracycline 7-halogenase | 1.14.19.49 | K14257 |
| tryptophan 7-halogenase | 1.14.19.9 | K14266 |
| ferroxidase | 1.16.3.1 | NA |
| bacterial non-heme ferritin | 1.16.3.2 | NA |
| xanthine dehydrogenase | 1.17.1.4 | NA |
| (light-dependent) protochlorophyllide reductase | 1.3.1.33 | NA |
| coproporphyrinogen oxidase | 1.3.3.3 | NA |
| 9,9’-dicis-zeta-carotene desaturase | 1.3.5.6 | NA |
| short-chain acyl-CoA dehydrogenase | 1.3.8.1 | NA |
| dihydroorotate dehydrogenase (fumarate) | 1.3.98.1 | NA |
| L-aspartate oxidase | 1.4.3.16 | NA |
| glycine oxidase | 1.4.3.19 | NA |
| D-amino-acid oxidase | 1.4.3.3 | NA |
| monoamine oxidase | 1.4.3.4 | NA |
| pyridoxal 5’-phosphate synthase | 1.4.3.5 | NA |
| nitrate reductase (NADH) | 1.7.1.1 | NA |
| ferredoxin-nitrate reductase | 1.7.7.2 | NA |
| cytochrome-c oxidase | 1.9.3.1 | NA |
| thymidylate synthase (FAD) | 2.1.1.148 | NA |
| 5-aminolevulinate synthase | 2.3.1.37 | NA |
| aralkylamine N-acetyltransferase | 2.3.1.87 | NA |
| sucrose-phosphate synthase | 2.4.1.14 | NA |
| protein O-GlcNAc transferase | 2.4.1.255 | NA |
| 15-cis-phytoene synthase | 2.5.1.32 | NA |
| 4-(cytidine 5’-diphospho)-2-C-methyl-D-erythritol kinase | 2.7.1.148 | NA |
| crossover junction endodeoxyribonuclease | 3.1.22.4 | NA |
| 3’,5’-cyclic-GMP phosphodiesterase | 3.1.4.35 | NA |
| phospholipase D | 3.1.4.4 | NA |
| DNA-3-methyladenine glycosylase II | 3.2.2.21 | NA |
| leucyl aminopeptidase | 3.4.11.1 | NA |
| glutamyl endopeptidase | 3.4.21.19 | NA |
| ribulose-bisphosphate carboxylase | 4.1.1.39 | NA |
| deoxyribodipyrimidine photo-lyase | 4.1.99.3 | NA |
| deoxyribodipyrimidine photo-lyase | 4.1.99.3 | NA |
| aldehyde oxygenase (deformylating) | 4.1.99.5 | NA |
| nitrile hydratase | 4.2.1.84 | NA |
| chorismate synthase | 4.2.3.5 | NA |
| DNA-(apurinic or apyrimidinic site) lyase | 4.2.99.18 | NA |
| lactoylglutathione lyase | 4.4.1.5 | NA |
| adenylate cyclase | 4.6.1.1 | NA |
| guanylate cyclase | 4.6.1.2 | NA |
| long-chain-fatty-acid—CoA ligase | 6.2.1.3 | NA |
| DNA ligase (ATP) | 6.5.1.1 | NA |
| DNA ligase (NAD+) | 6.5.1.2 | NA |
| cytochrome-c oxidase | 7.1.1.9 | NA |
| Flavodiiron (Flv1a/3a) | NA | NA |
