# Supplementary material for: Prochlorococcus marinus responses to light and oxygen
Source: PLoS One. 2024 Jul 22;19(7):e0307549. doi: 10.1371/journal.pone.0307549 (PMC11262661; doi:10.1371/journal.pone.0307549)
Supplement: S2 Table — (DOCX) [file pone.0307549.s007.docx]

| Strain | [O_2_] (µM) | µ_max_ (d^-1^) | Photoperiod (h) | PAR (µmol photons m^-2^ s^-1^) | Spectral waveband (nm) |
| --- | --- | --- | --- | --- | --- |
| MED4 | 250 | 0.68 | 16 | 180 | 450 |
| MED4 | 25 | 0.65 | 12 | 90 | 450 |
| MED4 | 2.5 | 0.00 | All tested | All tested | All tested |
| SS120 | 250 | 0.50 | 16 | 90 | White LED |
| SS120 | 25 | 0.45 | 8 | 90 | 450 |
| SS120 | 2.5 | 0.15 | 12 | 30 | 660 |
| MIT9313 | 250 | 0.54 | 16 | 30 | 450 |
| MIT9313 | 25 | 1.01 | 16 | 90 | White LED |
| MIT9313 | 2.5 | 0.45 | 12 | 90 | 450 |
